# Positive and strongly relaxed purifying selection drive the evolution of repeats in proteins

**DOI:** 10.1038/ncomms13570

**Published:** 2016-11-18

**Authors:** Erez Persi, Yuri I. Wolf, Eugene V Koonin

**Affiliations:** 1 National Center for Biotechnology Information, National Library of Medicine, National Institutes of Health, Bethesda, Maryland 20894, USA

## Abstract

Protein repeats are considered hotspots of protein evolution, associated with acquisition of new functions and novel phenotypic traits, including disease. Paradoxically, however, repeats are often strongly conserved through long spans of evolution. To resolve this conundrum, it is necessary to directly compare paralogous (horizontal) evolution of repeats within proteins with their orthologous (vertical) evolution through speciation. Here we develop a rigorous methodology to identify highly periodic repeats with significant sequence similarity, for which evolutionary rates and selection (d*N/*d*S*) can be estimated, and systematically characterize their evolution. We show that horizontal evolution of repeats is markedly accelerated compared with their divergence from orthologues in closely related species. This observation is universal across the diversity of life forms and implies a biphasic evolutionary regime whereby new copies experience rapid functional divergence under combined effects of strongly relaxed purifying selection and positive selection, followed by fixation and conservation of each individual repeat.

## Abstract (web summary)

Protein repeats may be considered a paradox, being evolutionarily conserved yet also hotspots of protein evolution associated with innovation. Here, the authors use a novel method to show that new repeats undergo rapid divergence within species, but are then fixed and conserved between species.

## Graphical abstract

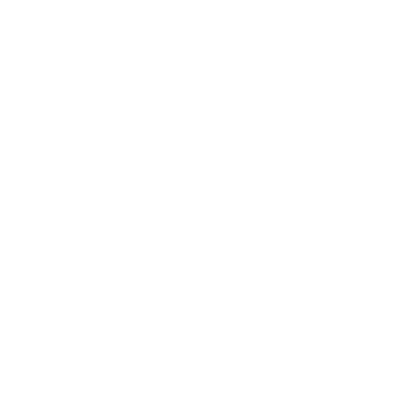

Numerous proteins in most life forms, but particularly animals and plants, contain compositionally ordered regions, which consist of recurring motifs, such as short tandem repeats, periodic structures and repetitive domains^1^,^2^,^3^,^4^,^5^. Hereinafter we refer to such recurring motifs simply as repeats. Repeats are crucially important, in particular, as building material for scaffolds of various macromolecular complexes, for example, nuclear pores^6^,^7^, the proteasome^8^ or mechanotransduction channels^9^. Examples of the most abundant repeats with scaffolding functions include ankyrin, tetratricopeptide (TPR) and WD40 repeats^10^,^11^,^12^,^13^,^14^,^15^. Repeats are also important in essential biochemical functions such as transcription regulation as exemplified by the extremely common Zn-finger repeats^16^,^17^.

Repeats can emerge by means of replication slippage and recombination^18^,^19^, grow into longer units^20^, and diverge by accumulating mutations. New repeats represent a major source of genetic variation, often associated with fast evolution and acquisition of new functions^21^,^22^,^23^. Striking examples, from diverse organisms, of the role played by gain and loss of protein repeats in microevolution include variation in the clock gene *period*, which is responsible for adaptation of the circadian clock to temperature in *Drosophila*^24^, the *Runx-2* gene, associated with morphological changes in dogs^25^, and cell wall proteins, leading to new cell adhesion phenotypes in fungi and protists, and thought to allow for evasion of the host immune system^26^.

Several comparative studies have shown that repetitive regions in proteins are globally conserved across species^27^,^28^,^29^,^30^, indicating that repeats are functional but also that fast evolution is rare^29^. Despite this strong evidence of the functionality and evolutionary conservation of repeats, repeat variation is also a known molecular driver of genetic disease^31^,^32^, which indicates the importance of rapid change in repetitive regions of proteins. Furthermore, rapid evolution of protein repeats plays key roles in various aspects of immunity as exemplified by the leucine-rich repeats, which are the key structural components of innate immunity proteins, such as animal Toll-like receptors and plant disease resistance proteins, as well as adaptive immunity components in jawless vertebrates^33^,^34^,^35^,^36^,^37^,^38^.

Thus, there seems to be a conundrum between the overall evolutionary conservation in repetitive regions of proteins and rapid change of repeats associated with a variety of biological processes. Here we resolve this apparent contradiction by revealing a dramatic difference between the regimes of intra-protein (horizontal) evolution of repeats and inter-protein (vertical) evolution of repeats in orthologous proteins.

To analyse the evolution of repeats and maximize the likelihood that evolutionary rates can be estimated, we develop a rigorous method to extract repeats with conserved length and significant sequence similarity from protein sequences. We validate it and apply it to systematically compare the horizontal and vertical evolution of repeats in diverse groups of organisms. We show that repeats are highly conserved between species, while horizontally propagating and diverging. Thus, each fixed repeat appears to be functionally important in itself and hence subject to purifying selection, whereas in the initial phase of the evolution of repetitive regions, a combination of strongly relaxed purifying selection and positive selection drives fast horizontal divergence of the repeat sequences, presumably yielding new functions. Because variation of repeats plays a crucial role in human disease, in particular neurodegeneration and cancer, the methodology employed here provides means to study somatic horizontal evolution of repeats, and could contribute to the identification of disease drivers associated with this mutational class.

## Results

### Methodology for identifying highly periodic repeats

**Figure 1:**
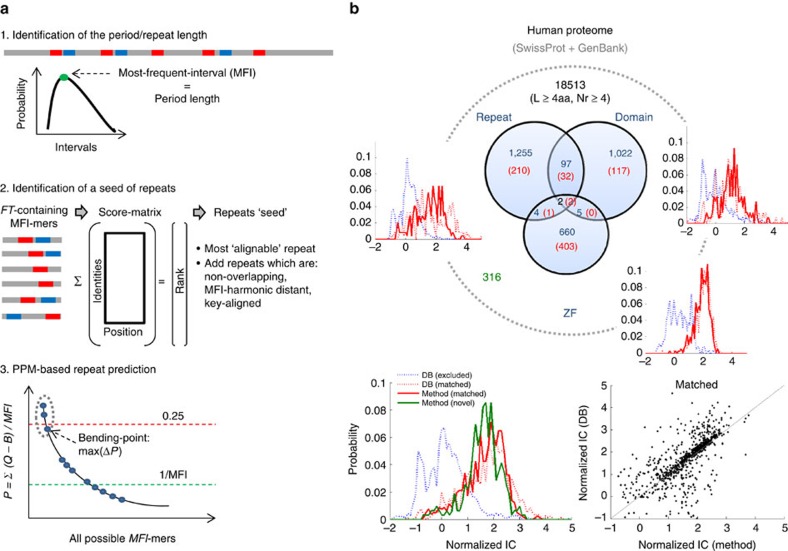
Three steps computational pipeline to extract repeats and its validation. (**a**) First, the period length (L) of repeats is inferred from the most frequent interval (MFI) of frequent triplets (FT). Second, a seed of repeats is identified by aligning all possible *k*-mers (*k=L=*MFI) that contain FT*s*, transforming the alignment into a scoring matrix, and selecting valid top ranked MFI-mers. Third, a probability position matrix (PPM), *Q,* is built from the seed over a background, *B,* to predict additional repeats. See Methods and Supplementary Methods for more details. (**b**) A set of 18513 canonical proteins from Swissprot that match information in GenBank, containing the corresponding coding DNA, are analysed. Swissprot annotates 3,045 proteins (blue numbers) which contain at least four repeats of length ≥4aa, assigned to 3 distinct classes (Repeats, Domains, Zinc-fingers). The method extracts a subset of 765 proteins (red) and identifies 316 novel ones (green), totaling 1,081 proteins with repeats. It excludes non-periodic and/or highly diverged repeats (blue dotted curves), and includes only repeats of identical length and high sequence similarity (red and green solid curves). Repeats in matched proteins (red numbers) have similar IC to the annotated repeats (red dotted versus red solid; and IC scatter subplot).

Numerous algorithms for repeat detection have been developed^39^. However, because repeats manifest rich patterns of recurrence, divergence and variable lengths, a single uniform detection methodology does not appear to be attainable, and each algorithm is tuned to identify specific patterns of the repetitive phenomena^40^. Most of the repeat-detection algorithms are not suitable for large-scale analysis^41^. Moreover, due to the relatively short lengths of repeats and their divergence, estimates of evolutionary rates are often unreliable. Nonetheless, for subsets of repeats that are highly periodic (that is, have identical length) and possess significant sequence similarity, evolutionary rates can be estimated in many cases and become meaningful when averaged across many comparisons, as long as there are no systematic biases. This is the approach employed in the present study. To this end, we develop a computational pipeline to rapidly extract ‘near perfect' repeats from protein sequences in a systematic manner and validate it against the well-annotated human Swissprot reference proteome (Fig. 1). Throughout this study, we focus on repeats which are at least four amino-acid long and which recur at least four times in a protein.

The method, illustrated in Fig. 1a, includes three main steps. First, the basic characteristics of the repeats, such as the period length and the region that encompasses most of the repeats, are determined from the distribution of frequent triplets (FT) in a protein, following the compositional order approach^4^. By relying on triplets, diverged repeats can be identified, as long as the periodic structure is significant. Second, several well-defined repeats are identified and serve to build a seed, as follows: all possible repeats (that is, all possible *k*-mers, which contain FTs, where *k* equals the period length, *L*) are extracted, aligned, transformed into a scoring matrix and ranked. The repeat that is most ‘alignable' with all other possible repeats is identified first, and as such determines the best choice of the exact locations of repeats, with the ultimate goal to maximize the overall normalized information content (IC) of the aligned repeats, over all amino acids and positions (IC*=*1/*L∑∑*IC_*ij*_, *i=*1−20, *j=*1−*L*). Then, more repeats are added to the seed if they: (i) contain a key, a triplet that recurs the most within the period, and are key-aligned; and (ii) are separated from each other by a distance that is equal to the period length or its harmonic. These properties ensure that all seed repeats are parts of the periodic structure. Third, based on the seed, a probability position matrix is defined over a background^42^, and additional repeats are predicted by scanning the entire protein (see Methods and Supplementary Methods for additional details). Under this procedure, by design, all repeats are of identical length and possess detectable sequence similarity to each other; copies that contain insertions or deletions or are highly diverged (for example, most WD repeats) are discarded.

Figure 1b illustrates the validation of our repeat detection method by comparing it with Swissprot annotations of human proteins. Swissprot annotates repeats based on various methodologies, including the traditionally used REP algorithm^43^, and assigns the majority of repeats (>96%) to three distinct classes (Simple short repeats, repetitive domains and Zn-fingers). As shown, the method excludes repeats, of any class, which have poor IC, detects only the repeats with high IC and predicts ∼10% novel repeat-containing proteins with high repeat IC (see example in Supplementary Fig. 1). It has to be emphasized that this method is not intended to be the optimal among existing algorithms, but to allow for a systematic large-scale comparative study, of a maximal number of high IC repeats, across diverse organisms.

### Application of the methodology for single protein analysis

**Figure 2:**
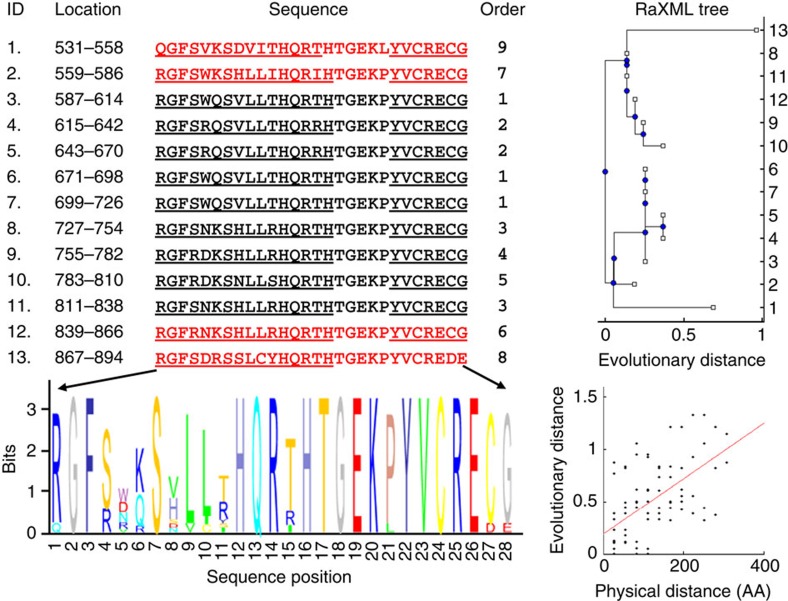
Example of analysis of a single protein, the 894AA long human zinc-finger *PRDM9*. The 13 tandemly 28AA long repeats (ID) are identified by the algorithm at the end of the protein, ordered by their location on the protein. Underlined letters correspond to the Zinc fingers annotated in SwissProt (first finger starts 7AA before the beginning of the first repeat). The order by which the method accumulates the repeats (Order), reveals clusters of identical repeats: 1 (ID=3,6,7), 2 (ID=4,5) and 3 (ID=8,11). Black coloured repeats represent the seed identified in the second step, and red coloured repeats are those identified by the PPM-based predictor in the third step. Repeats are highly similar, with high IC, as shown by the sequence logo. A maximum-likelihood tree of the repeats is shown in the right panel, where the repeats IDs are given on the *y* axis. The plot beneath the tree shows the positive correlation between evolutionary distance (in substitutions per site) and physical distance (in amino acids), obtained by comparing all repeat pairs, where the red line represents a linear regression fit (Spearman correlation=0.53, *P* value=6.7e−7). The mean d*N/*d*S* for all pair comparisons (*n=78*): *<*d*N/*d*S>*=2.7146±0.23, that is, significant positive selection.

Figure 2 exemplifies the analysis of a single protein, the human *PRDM9* Zn-finger DNA-binding protein, which contains 13 tandem repeats. Additional examples are provided in Supplementary Figs 1 and 2, emphasizing that repeats can form diverse patterns and do not always recur in perfect tandem. The *PRDM9* protein binds to double-stranded DNA breaks and promotes meiotic recombination in humans and mice, and is the only mammalian gene so far shown to play a distinct role in speciation^44^,^45^. Rapid evolution of *PRDM9* has been demonstrated including lineage-specific expansion of the Zn-fingers and positive selection in DNA-contacting positions^46^,^47^. With the sequences of repeats at hand, we represent their evolutionary relationships by a maximum-likelihood repeat tree (see Methods), from which the evolutionary distances between repeats can be estimated and compared with the respective physical distances. Furthermore, treating repeats as paralogous elements, we estimate their pairwise d*N/*d*S* (synonymous to non-synonymous substitution rates) ratios by comparing the coding sequences for each pair of repeats (Methods). The mean over all pairs, *<*d*N/*d*S>*, yields a stable measure, as indicated by the small error on *<*d*N/*d*S>*, for the horizontal evolution of repeats within a protein. In the case of *PRDM9*, <d*N*/d*S>*=2.7±0.2, which is unequivocal evidence of positive selection in the horizontal evolution of the Zn-finger repeats, in agreement with previous findings^47^. We next apply this analysis to all 1081 repeat-containing proteins identified in Swissprot (Fig. 1b).

### Horizontal evolution of repeats in the human proteome

**Figure 3:**
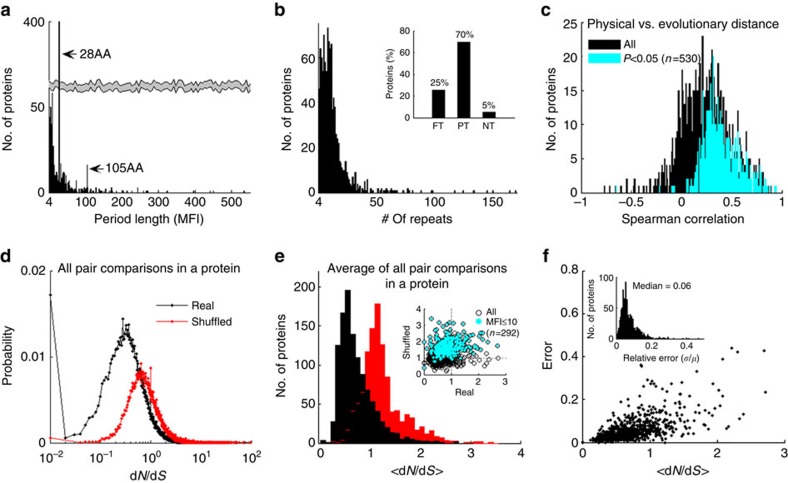
Repeats in the human proteome. (**a**) Distribution of the most frequent interval (MFI) representing the period/repeats length. (**b**) Distribution of the number of repeats in a protein. Inset depicts the distribution of recurrence type (FT=fully tandem; PT=partially tandem, that is, at least two repeats recurring in tandem; NT=non-tandem). See examples in Supplementary Fig. 1. (**c**) Spearman correlation between the physical and evolutionary distances of repeats in a protein. (**d**) Distribution of d*N/*d*S* ratios of all valid pair comparisons in a protein, across the proteome, on log-scale (*n=*319K, out of all possible 510K pairwise comparisons). Black curve corresponds to the identified repeats (median=0.52) and red curve corresponds to shuffled repeats (median=0.99). Bins equal 0.01. (**e**) Distribution of the mean d*N/*d*S* ratio of all valid pair comparisons in a protein, *<*d*N/*d*S>*, across the proteome, for real repeats (black) and shuffled repeats (red). Bins equal 0.1, shown on linear-scale. Inset shows a scatter plot of *<*d*N/*d*S>* of real repeats versus the respective shuffled repeats, where very short repeats (≤10AA) are superimposed (cyan). (**f**) The relationship between *<*d*N/*d*S>* and the error on the mean. Inset depicts the relative error.

The statistics of the repeats and their evolutionary characterization across the human proteome are summarized in Fig. 3 (Supplementary Data 1). The distribution of the repeat lengths (Fig. 3a) highlights evident peaks observed at: MFI*=28AA,* identified in 37% of the proteins (398 of 1,081), all of which are Zn-fingers, and at *105AA,* associated with protocadherin repeats. Other dominant families are keratin, collagen, and ankyrin repeats. Enrichment analysis of GO annotations, using GOrilla^48^, shows that functional categories DNA/RNA binding, transcription, regulation, extracellular organization, and various metabolic and biosynthesis processes are enriched for proteins containing repeats (Supplementary Data 2).

Typically, human proteins contain 10 to 30 repeats (Fig. 3b). In 25% of the repetitive proteins, all repeats recur in tandem, and in 70% of the proteins, repeats partially recur in tandem, that is, there are at least two tandem repeats whereas others are interspersed (see examples in Supplementary Fig. 1). Although maximum-likelihood trees of repeats in each protein are not highly reliable due to the small size of the repeats, analysis of such trees across the proteome reveals a highly significant positive correlation between the physical and evolutionary distances, whereas a significant negative correlation is rarely observed (Fig. 3c). These observations indicate that physically adjacent repeats tend to be similar and by inference evolutionarily related. Thus, the horizontal dynamics of repeats appears to be governed by tandem duplication followed by sequence divergence due to accumulation of mutations, in accordance with previously described mechanisms^18^,^20^.

To further characterize the horizontal evolution of repeats, we directly assess the protein-level selection (d*N/*d*S*) of all repeat pairs within each protein, as in Fig. 2, in all proteins. The distributions of d*N/*d*S* values in human protein repeats, compared with randomized repeats, are shown in Fig. 3d–f. About a third of the pairs were discarded from the analysis because the respective repeats were too short and/or too far diverged, such that either d*N* or d*S* could not be measured (Methods). The distribution of d*N/*d*S* values for all valid pair comparisons within proteins across the proteome shows that, for an overwhelming majority of the comparisons, d*N/*d*S*<1 and is substantially smaller compared with the d*N/*d*S* values for the respective randomized repeats that were generated by shuffling the coding DNA sequences of the real repeats (Fig. 3d). This observation holds also for the mean d*N/*d*S* over all pairwise comparisons within a protein (*<*d*N*/d*S>*; Fig. 3e). The small relative errors of *<*d*N/*d*S>* indicate that it is a fairly stable estimate, despite the short lengths of the repeats (Fig. 3f).

The long tails in the distributions of d*N/*d*S* and *<*d*N/*d*S>* (Fig. 3d,e) suggest that horizontal evolution of the repeats in some proteins involves positive selection (that is, d*N/*d*S*>1). Significant positive selection can be detected by requiring that *<*d*N/*d*S>* of real repeats would be substantially greater than that of the respective randomized repeats, and this is indeed the case for numerous proteins (Fig. 3e). This is a strict requirement because, unlike in real data, *<*d*N/*d*S>* of randomized short repeats is much greater than that of longer repeats, as can be expected from small-number statistics (Fig. 3e, inset). This observation further testifies to the stability of *<*d*N/*d*S>* in real data by showing that it is only weakly sensitive to the repeat length. Notably, the *PRDM9* gene discussed above (Fig. 2), which is involved in meiotic recombination and speciation, shows the highest <d*N/*d*S*> value for the horizontal comparison of repeats among all human protein-coding genes. Analysis of the GO enrichment in proteins ranked by <d*N/*d*S>* shows that high <d*N/*d*S>* values are associated with chromatin, nucleosome and cellular organization; DNA metabolism; nucleoside phosphate binding; nucleotide and RNA binding; and various metabolic functions (Supplementary Data 3).

### Universal patterns of repeat evolution in diverse organisms

**Figure 4:**
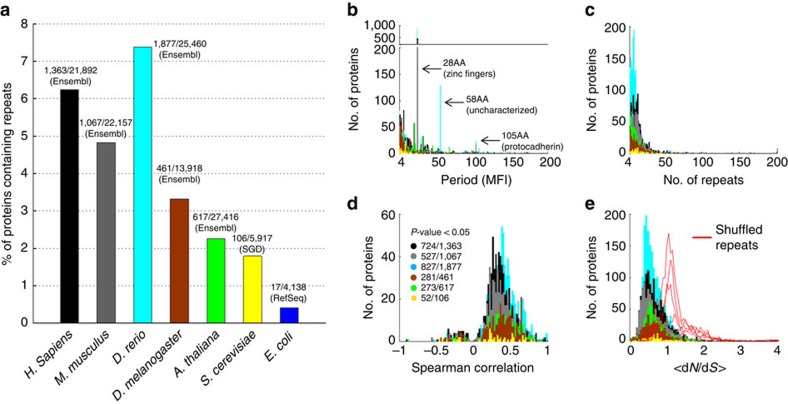
Protein repeats in diverse organisms. (**a**) Percentage of proteins containing repeats in the studied organisms. Species are arranged by taxonomic order. The colour code, the number of proteins containing repeats relative to the number of unique genes and database sources are shown. (**b**) Distribution of the period lengths demonstrating similarities and differences between the organisms. (**c**) Distribution of the number of repeats in each organism. (**d**) Distribution of the Spearman correlation coefficients between evolutionary and physical distances of the repeats in each protein. (**e**) Distribution of *<*d*N/*d*S>* values for horizontal comparisons of repeats from different organisms (colour coded) compared with the respective distributions of shuffled repeats (red curves).

**Figure 5:**
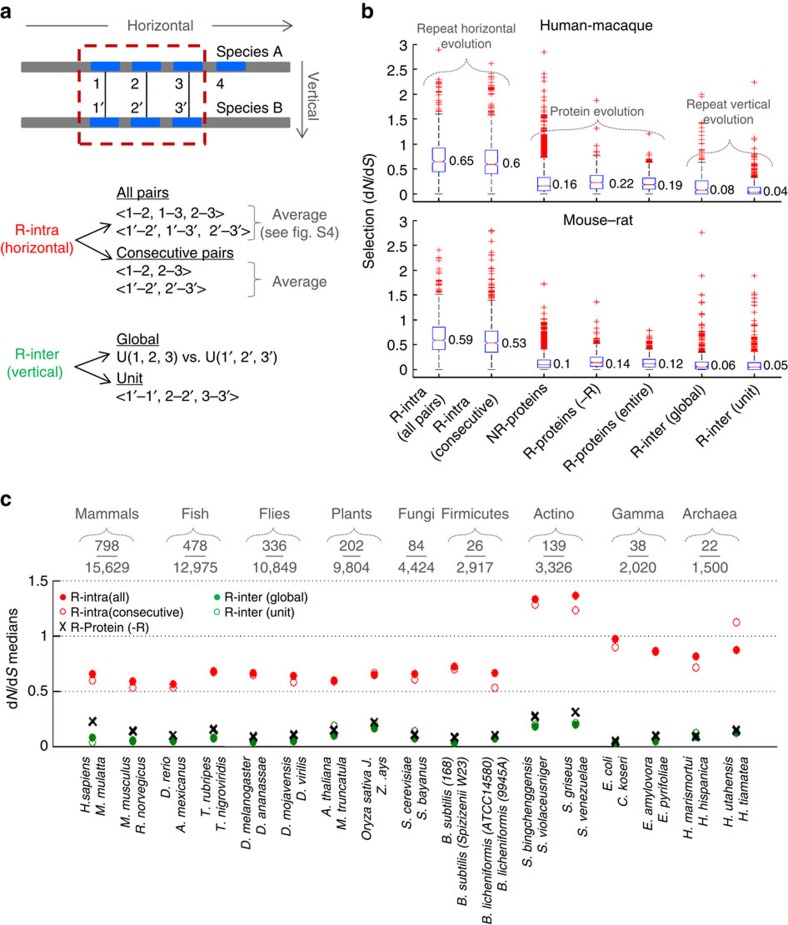
Selection in the horizontal and vertical evolution of repeats in sets of orthologous proteins. (**a**) Schematic illustration of two aligned orthologous proteins from species A and species B, containing 4 and 3 repeats (marked as blue segments), respectively. Both horizontal and vertical selection measures are based on all orthologous repeats (indicated by the dashed box). Horizontal selection (R-intra) is given by the average across species of *<*d*N/*d*S>* (Supplementary Fig. 4), where *<*d*N/*d*S>* is estimated twice: once as the average d*N/*d*S* of all repeat pairs within a protein/species, and second as the average d*N/*d*S* of all consecutive pairs of repeats. Vertical selection (R-inter) is estimated, once globally, by the d*N/*d*S* of merged segments of orthologous repeats, and locally (unit-based), by the average d*N/*d*S* of all orthologous repeat pairs. (**b**) Distribution of d*N/*d*S* for the horizontal evolution of repeats in repeat (R) containing proteins (*n*=798), followed by the distribution of d*N/*d*S* of complete proteins: non-repetitive (NR) proteins (*n*=10,086), and repetitive proteins, with (entire) or without (−R) the repetitive part, followed by d*N/*d*S* distributions for the vertical evolution of repeats. (**c**) The medians of the distributions in **b** for all pairs of organisms within each quartet or pair of species. At the top, the numbers of orthologous repetitive proteins relative to the number of all orthologous proteins in each quartet or pair are shown. For the complete d*N/*d*S* distributions of all species pairs, see Supplementary Fig. 8.

Next, we similarly analyse a set of organisms from several diverse major taxa (Fig. 4). As expected, the number of repetitive proteins significantly drops from vertebrates to invertebrates to plants to unicellular organisms (Fig. 4a). There are both evident similarities and differences in the distributions of the period lengths (Fig. 4b). Zn-fingers are ubiquitous in vertebrates, but not in other organisms. In fish, there is a large family of uncharacterized repetitive proteins with period of 58AA. The dominant periods in the fly (*Drosophila melanogaster*) are 6AA (*n=*62), associated with a variety of functions and some uncharacterized proteins (*n=*37), and 18AA (*n=*27), mostly in histone H1. In plants (*Arabidopsis thaliana*), the dominant period is 24AA (*n=*57), associated with diverse functions. In yeast (*Saccharomyces cerevisiae*), the dominant period is *12AA,* associated with Helicases, and additionally, there is a clear enrichment of cell wall proteins (25 of 106 among repetitive proteins compared with 215 of 5,917 in the entire proteome) with various period lengths. Despite these notable differences between the functional repertoires of repetitive proteins, both the horizontal propagation of repeats within proteins implied by the correlation between physical and evolutionary distances (Fig. 4d) and the much lower *<*d*N/*d*S>* values for the horizontal evolution of repeats compared with shuffled repeats (Fig. 5e) are universal phenomena.

Taken together, these results indicate that horizontal evolution of repeats within proteins is subject to purifying selection (d*N/*d*S*<1). However, the high level of divergence among repeats in many proteins, along with the presence of heavy tails in d*N/*d*S* value distributions, raises the question how much of this divergence is a consequence of relaxed purifying selection and how much could be caused by positive selection. To address this question, we turn to the analysis of orthologous sets of proteins across diverse life forms and compare the horizontal evolution of paralogous repeats with the vertical evolution of orthologous repeats in closely related species.

### Fast horizontal diversification versus vertical conservation

We apply the repeat analysis methodology to sets of orthologous proteins in quartets or pairs of species starting with a well-annotated mammalian species quartet: human, macaque, mouse and rat (Fig. 5). The number of identified repeats can vary among species. We assume that this variability, Rvar, should be minimal between closely related species and therefore use it as a criterion to remove horizontally divergent repeats such that the overall IC, across all repeats in all orthologues, is maximized (Supplementary Fig. 3). Among the 15,649 proteins represented by orthologues in all 4 species, we identified 798 proteins that contain at least 4 repeats in each of the 4 species.

For each orthologous protein pair, we assess selection for: (i) horizontal evolution of repeats, and (ii) vertical evolution of the respective orthologous repeats (see illustration in Fig. 5a). Horizontal selection (R-intra) is estimated in two ways: first, by the mean d*N/*d*S* of all repeat pairs within a protein, and second, given the evolutionary proximity of physically adjacent repeats (Figs 3c and 4d), by the mean d*N/*d*S* of all consecutive repeat pairs. The mean horizontal selection across repeat pairs, *<*d*N/*d*S>*, is highly correlated between orthologues in closely related species (Supplementary Fig. 4) indicating that similar mechanisms affect horizontal evolution of repeats across species. Thus, we use the simple average of *<*d*N/*d*S>* across two species as a single selection measure of horizontal evolution of repeats for an orthologous protein pair. For the analysis of the vertical evolution (R-inter), we measure d*N/*d*S* by comparing globally the repetitive regions of orthologous proteins, as well as by comparing each pair of orthologous repeats separately. The obtained distributions are then compared with the whole-protein d*N/*d*S* values of the respective proteins, with and without the repetitive regions, as well as with the d*N/*d*S* values of non-repetitive proteins (Methods).

The comparison of these d*N/*d*S* distributions (Fig. 5b,c) shows that horizontal evolution of paralogous repeats is substantially accelerated compared with the vertical evolution of the respective orthologous repeats. In accordance with their likely origin by duplication, adjacent repeats show slightly but significantly lower d*N/*d*S* values than the mean for all repeats. This accelerated horizontal evolution of repeats is independent of the selection acting on the entire protein or its non-repetitive part (Supplementary Fig. 4), suggesting effects of evolutionary forces acting horizontally on repeats beyond the dominant purifying selection acting vertically on proteins. Vertically, repeats are at least as conserved as the proteins in which they reside. Often, the mean d*N/*d*S* of repeats is lower than that of the respective complete proteins, indicative of strong purifying selection on orthologous repeat units. Moreover, as shown by the measurements of the average strength of selection affecting each unit of orthologous repeats, each individual copy of a repeat is highly conserved across species. These findings reveal a dramatic difference between the selection regimes of horizontal and vertical evolution indicating that, although in most of the comparisons d*N/*d*S*<1, horizontal evolution of repeats seems to drive them apart, leading to substantial divergence of repeat copies within individual proteins. This divergence contrasts the strong vertical conservation across species, suggesting that not only is purifying selection strongly relaxed once new repeats are generated, but that many repeats are positively selected during evolution following their generation, presumably with functional consequences (Discussion). After a period of strongly relaxed purifying selection (and/or positive selection), the unique functions of the individual repeats appear to be fixed causing a substantial slowdown of vertical evolution.

### Universality of the accelerated horizontal evolution

Testing other sets of orthologues from species quartets or pairs across the diversity of life forms, including fish, flies, plants, fungi, bacteria and archaea, we establish that the fast horizontal evolution of repeats contrasting their evolutionary conservation between species is a universal phenomenon (Fig. 5c). The variability of the results is greater in prokaryotes compared with eukaryotes because the fraction of repetitive proteins in prokaryotes is low. It is of interest, however, that in actinobacteria, bacteria with complex cellular organization^49^ that possess more repetitive proteins than other prokaryotes; the horizontal divergence of repeats is the highest among all analysed organisms, resulting in a particularly dramatic difference between horizontal and vertical evolution.

Because of this marked effect and the short lengths of repeats, we test the sensitivity of the results to possible effects of saturation in synonymous or non-synonymous substitutions (see Methods for details) and find that these results are highly robust, and free of detectable biases for repeats longer than 10AA (Supplementary Fig. 5). Furthermore, the results are robust to the particular choice of the method used to estimate d*N/*d*S* (Supplementary Fig. 6), but because of the short lengths of sequences compared, we chose the least-parametrized method for the bulk of the analysis (Methods). Finally, we test whether the evolutionary age of repeats has any systematic effect that could bias our d*N/*d*S* estimates. Both vertically and horizontally, the apparent antiquity of proteins or repeats is uncorrelated with the d*N/*d*S* estimates (Supplementary Fig. 7). This robustness is expected given the similarity of the d*N/*d*S* distributions across the diversity of eukaryotes (Fig. 5c; Supplementary Fig. 8). Hence, the high horizontal d*N/*d*S* estimates are unlikely to be a consequence of evaluation across duplication events that, by definition, predate the split of the respective species (in a quartet), compared with vertical evolution that is evaluated over shorter evolutionary time scales.

### Propagation of beneficial mutations in repeat arrays

To further explore the dynamics of repeats, we assess, from the maximum-likelihood trees of repeats within a species quartet (see Methods and example in Supplementary Fig. 2), the extent of their homogenization in each protein and each species, and test the possible contribution of horizontal selection to this process (Supplementary Figs 9 and 11). Significant homogenization, that is, presence of two or more paralogous repeats in a given protein that are more similar to each other than to their counterparts in orthologous proteins from other species, can be considered evidence of molecular drive leading to concerted evolution, by analogy to the fate of duplicated genes (Supplementary Fig. 9). Three principal molecular drives have been proposed as underlying mechanisms leading to homogenization of tandemly repeated sequence arrays^50^,^51^: (i) duplication, that is, amplification of a single unit, (ii) recombination, in particular unequal crossing-over and gene (biased) conversion, which can spread the mutations occurring in a repeat, and (iii) strong selective pressure, in the form of either purifying selection, which homogenizes the repeats by eliminating deleterious mutations, or positive selection which propagates beneficial mutations, thus leading to homogenization.

We find that significant homogenization of the repeats, in at least one of the species, is observed in ∼30% of the repetitive proteins (Supplementary Fig. 10). Nonetheless, in the majority of orthologous proteins that contain identical numbers of repeats (Rvar=0), the paralogous repeats have diverged further than each of them diverged from the respective orthologues, that is, the repeats are not homogenized. These findings imply that in most cases where homogenization is detectable, it is a consequence of a mechanism that generates repeat variability (Rvar>0). Such a mechanism is consistent with recent duplication of new identical copies, following a ‘birth-and-death' process that presumably occurs independently in each species, and therefore leads to variability in the number of repeat across species. Under this model, new copies simply did not have time to evolve, and therefore, appear homogenized with no apparent functional consequence. Further evidence of the contribution of recent duplication to repeat homogenization comes from the observation that repeats are more homogenized at the clade level compared with the species level. Thus, repeats can be inferred to have been more similar to each other in each common ancestor, that is, soon after duplication, than they are in the extant species. All these observations hold throughout the diversity of the analysed organisms (Supplementary Fig. 11). Only in a few proteins, where the level of homogenization is high and the repeat variability, Rvar, is low, can one confidently infer that other mechanisms than recent duplication lead to concerted evolution.

Notwithstanding the relative rarity of homogenization, further analysis of the relationship of the homogenization levels of repeats between orthologous proteins reveals two distinct patterns (Supplementary Fig. 10): in some proteins, repeats are homogenized in only one of the orthologues, that is, homogenization is species-specific, whereas in other proteins, the homogenization levels are significantly correlated between the two orthologues, that is, appear to be protein-intrinsic. Species-specific homogenization of repeats in a protein can be viewed as a further indication of independent duplication, but the positive correlation of the repeat homogenization levels between orthologous proteins implies that homogenization is a consequence of a functional molecular drive that acts at the protein level and is presumably important in both species. This correlation is detectable across the diversity of the analysed life forms (Supplementary Fig. 11). In an attempt to elucidate the nature of this putative additional molecular drive, we assess the relationship between repeat homogenization and horizontal selection (Supplementary Fig. 10). A weak but significant (in vertebrates and plants) positive correlation is detected between repeat homogenization and d*N/*d*S* values (Supplementary Fig. 11). This correlation suggests that the high horizontal d*N/*d*S* values typical of the horizontal evolution of repeats reflect (at least in part) positive selection that contributes to repeat homogenization by horizontal propagation of beneficial mutations through the repeat array and thus constitutes a selective molecular drive.

## Discussion

Repeats in protein-coding genes are often viewed as a major source of new raw genetic material, which rapidly evolves and facilitates the acquisition of new functions and complex phenotypic traits^22^,^23^,^24^,^25^,^26^,^52^. Under this view, a new copy is assumed to be free of selective constraints, such that a new function can emerge rapidly, minimizing the risk of deleterious effects. However, the global conservation of repeats across species^27^,^28^,^29^ puts into question the concept of rapid evolution and the above neofunctionalization scenario. In other words, analogously to the fate of duplicated genes^53^, these observations reflect Ohno's dilemma^54^,^55^, which projects here to paralogous elements within proteins: if repeats are subject to strong purifying selection to maintain a function, how can they rapidly diverge?

**Figure 6:**
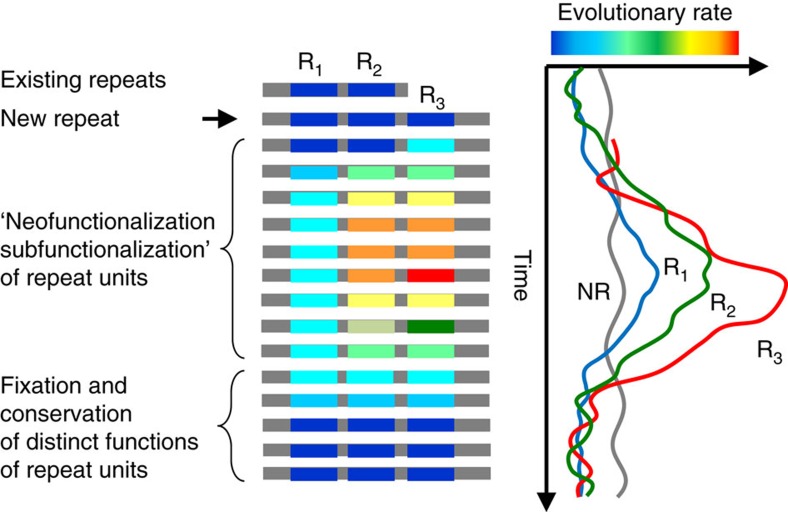
A model of repeat evolution. Existing repeats (R1, R2) are functional and conserved. Once a new copy is generated (R3), evolution of all copies starts to accelerate. This acceleration is presumably more pronounced for the new copy, and those physically adjacent to it (see colour scale of the evolutionary rate). During this phase, the new copy (and possibly others) can acquire a new function (neofunctionalization) or become specialized towards improved execution of one of the existing functions (subfunctionalization). This phase is dominated by strongly relaxed purifying selection and positive selection, resulting in substantially increased evolutionary rates of R1, R2 and R3 (right panel). Once the new functions are fixed, evolution of the repeats slows down to rates characteristics, or even below, of the non-repetitive (NR) part of the protein (grey), and the repeats are maintained presumably due to strong purifying selection.

For duplicated genes, it has been shown that, although subject to purifying selection (d*N/*d*S*<<1), they evolve slightly but significantly faster than orthologous genes of similar divergence^56^,^57^. Thus, the paralogues experience a phase of relaxed purifying selection shortly after duplication. Similarly, here we show that orthologous repeats evolve under selection that is, on average, at least as strong as the selection on the non-repetitive parts of protein sequences if not somewhat stronger, in agreement with previous studies. Importantly, we show that this selection acts on each copy, clearly indicating that individual repeats possess unique functions maintained by selection. However, within a protein, paralogous repeats diverge from each other substantially (typically, by an order of magnitude) faster than orthologous repeats as indicated by high d*N/*d*S* values characteristic of the horizontal evolution of repeats. On average, the horizontal d*N/*d*S* values, although much higher than the vertical values, are below unity, suggestive of strongly relaxed purifying selection. Nonetheless, the presence of long tails in the d*N/*d*S* distributions and especially the homogenization of repeats in some proteins that is apparently caused by selective drive suggest that positive selection also is involved in the horizontal evolution of repeats. Taken together, these observations translate into a general scenario for the evolution of repetitive regions in proteins that involves an initial phase of rapid sequence and functional diversification, driven in part by positive selection, following a burst of duplication, which leads to the emergence of a repetitive region, with subsequent fixation of the sequences of individual repeats and their ensuing slow evolution under purifying selection (Fig. 6).

This scenario resembles existing models for the evolution of duplicated genes^57^, but with important quantitative differences, which seem to translate into a qualitative distinction. The early acceleration of evolution in duplicated genes is relatively small and takes advanced analysis to be demonstrated convincingly^53^,^56^. Primarily, this acceleration among newly duplicated gene copies is attributed to subfunctionalization whereby duplicates quickly diverge under relaxed purifying selection, while losing some of the multiple functions of the ancestral gene^57^,^58^. The present analysis demonstrates a much greater difference in the divergence rates during horizontal versus vertical evolution of repeats. This finding implies a different regime for the evolution of repetitive regions in proteins where subfunctionalization could make some contribution but neofunctionalization and positive selection appear to be important, if not key factors. This distinction between the evolutionary regimes of paralogous genes and within-gene repeats could have to do with the small size of the latter whereby even a single amino-acid substitution would often cause a functional change of sufficient consequences to drive purifying or positive selection. After the initial accelerated evolution phase, the evolutionary trajectories of duplicated genes and protein repeats appear to be similar, that is, dominated by (often strong) purifying selection. Extending this analogy, our results indicate that evolution of most repetitive regions in proteins occurs via the straightforward birth-and-death (duplication and loss) route. However, we also found evidence of some concerted evolution, apparently via propagation and homogenization of the duplicated elements driven by positive selection. Although concerted evolution mostly occurs through DNA repair and replication mechanisms, such as unequal crossing over and (biased) gene conversion, the role of strong positive selection, allowing beneficial mutations to propagate, has been noticed as a contributing factor as well^19^,^59^. The existence of this form of drive implies that the observed high d*N/*d*S* values for the horizontal comparisons of repeats at least in part reflect positive selection that drives propagation of beneficial mutations across a repeat array.

The evolutionary relevance of the model presented here is likely to extend beyond readily detectable repeats, as suggested by the robustness of the horizontal and vertical selection estimates with respect to the IC of the repeats (Supplementary Fig. 12). This scenario, with its initial phase dominated by positive selection, potentially describes a major route of innovation in protein evolution that could apply to many protein families in which repeats are not easily detectable or at least are not conducive to straightforward analysis of the type described here, due to their high divergence. Furthermore, this route of evolution could have been particularly important for major expansions of the proteome at evolutionary transitions, such as the origin of eukaryotes^22^. However, it should be noted that for highly diverged repeats, such as those identified by HHrepID^60^, where estimates of d*N/*d*S* are often unattainable, the net effect of selection might be closer to neutrality (Supplementary Fig. 12). Furthermore, the biphasic evolutionary model formulated here represents a general trend in repeat evolution that might have limited applicability to certain repeat families, for example, helical repeats, such as HEAT or ARM, in which the function of protein binding is dispersed among multiple repeat units^61^.

The results of the present analysis emphasize the potential importance of the evolution of protein repeats in human disease, where horizontal expansion of repeats could play an adaptive role. In particular, cancer is marked by genomic instabilities, which can lead to duplications at all scales, from runs of amino acids to whole-genome duplications, and there is increasing evidence for the existence of a wide spectrum of complex insertion or deletions^62^. These instabilities generate inter- and intra-tumour heterogeneity, which enhances the capacity of cancers to progress, invade and metastasize^63^. Therefore, protein repeats could be especially important in cases of microsatellite instability^64^, where repeat dynamics is likely to occur, or when repeat unit variability occurs in a specific protein, as in the case of MUC1 (ref. 65). More generally, protein repeat dynamics in cancer is probably not fully characterized, due to known NGS assembly difficulties with repetitive sequences. Hence, somatic repeat expansion in cancers could be currently overlooked but potentially might comprise an important class of fast evolving drivers. The conceptual framework described here sets ground for quantifying this evolutionary adaptive path in tumours.

## Methods

### Data sets

Proteomes and their respective coding DNA of quartets of Mammals (*Homo sapiens*, *Macaca mulatta*, *Mus musculus* and *Rattus norvegicus*), Fish (*Danio rerio*, *Astyanax mexicanus*, *Takifugu rubripes* and *Tetraodon nigroviridis*), Fly from the *Drosophila* genus (*D. melanogaster*, *D. ananassae*, *D. mojavensis*, *D. virilis*) and Plants (*Arabidopsis thaliana*, *Medicago truncatula*, *Zea mays*, *Oryza sativa*; Japonica) were downloaded from Ensembl (V83). Quartet sets of prokaryotes: Gammaproteobactria (*Escherichia coli*, *Citrobacter koseri*, *Erwinia amylovora*, *Erwinia pyrifoliae*), Firmicutes from the Bacillus genus (*Bacillus subtilis*-168, *B. subtilis spizizenii* W23, *B. licheniformis* ATCC-14580 and *B. licheniformis* 9945A), Actinobacteria from the Streptomyces genus (*Streptomyces bingchenggensis* BCW-1, *S. violaceusniger* Tu-4113, *S. griseus* NBRC-13350, *S. venezuelae* ATCC-10712) and Archaea (*Haloarcula marismortui*, *Haloarcula hispanica*, *Halorhabdus utahensis* and *Halorhabdus tiamatea*) were downloaded from NCBI. Quartets were chosen such that they contain two sets of closely related species (for example, Human–Macaque and Mouse–Rat) and a larger evolutionary distance between the pairs (for example, primates and rodents), forming a more or less symmetric binary tree (Supplementary Fig. 9). This was done based on the evolutionary distances between protein sequences RNA polymerase II (POLR2A in *H. sapiens*, RpoC in *E. coli*) with the aim to find organisms with ∼95% sequence identity between closely related species, and ∼90% of sequence identity between species belonging to the two different clades of a quartet. For Fungi, we were unable to identify such a tree from the data at SGD, and therefore only a pair of species of Saccharomyces (*S. cerevisiae* and *S. bayanus*) was considered. Except for quartets from Ensembl, where predetermined orthologous sets are available^66^, we establish orthology using reciprocal best matches between well-alignment sequences (if the Needleman–Wunsch alignment score between sequences a and b is S_ab_, pairs with S_ab_/min(S_aa_,S_bb_)<0.1 were removed).

For single species proteome analysis, all unique proteins were included, taking the longest variant for each protein. Human proteome was analysed twice, once based on the reference proteome from SwissProt containing canonical sequences, complemented with the corresponding coding DNA sequences from GenBank, leading to 18,513 sequences for which we had both sequences (that is, proteins and their coding DNA), and second, based on Ensembl, from which we extracted ∼22K sequences. For all the data, from all sources, we verified that the coding DNA is exactly translated to the protein sequences.

### Computational pipeline of repeat identification

We employ a three-step computational pipeline to extract repeats from a protein sequence (Fig. 1). The first step identifies the region that contains a periodic structure and the length of the repeats composing it using the compositional order methodology^4^, which relies on the statistics of FTs, those that recur at least 5 times within a window of 2000 amino acids. The FTs are primarily distributed in low-complexity regions and periodic structures; hence, unless a periodic structure is insignificant, for example, composed of highly diverged elements, many of the triplets composing it will be assigned as FTs. From the distribution of FTs it is then possible to identify the length of the repeats as the MFI, that is, the maximum of the distribution of all intervals between two consecutive recurrences of the same FT. Obviously, in the absence of a periodic structure MFI is not defined because no interval of any FT recurs. Thus, the output of this step is the MFI (that is, the period length, which also represents the repeats length) and a key, which is the triplet that recurs most within the period, and serves as a handle for repeat alignment in the next step. Note that when a set of orthologous proteins is processed, the analysis of this step is based on the distribution of all FTs in all proteins, hence relies on a larger and more reliable statistics (see Supplementary Methods for more details).

In the second step, knowing the length of the repeats and the region in which the repeats are likely to reside, a seed of repeats is identified by extracting all possible MFI-mers in the protein, that is, *k*-mers with *k*=MFI with a sliding window of one amino acid, and aligning those that contain FTs. The aligned MFI-mers are transformed into a score-matrix that for each amino acid in a given position, counts the number of amino acids that are identical to it in all the other MFI-mers at this position. The sum of the scores across all positions ranks the MFI-mers. MFI-mers that are part of the periodic structure will be better aligned with each other than any other set of MFI-mers, and will be therefore top ranked. By definition, the top ranked MFI-mer is the most ‘alignable' and is the first to join the seed of repeats. Note that this is the best choice for determining the actual position of repeats, which is also expected to lead to IC maximization. Then, more repeats from the list of the FT-containing MFI-mers can be added to the seed if they are separated by a distance that is a multiple of MFI and are key-aligned.

In the third step, the entire protein sequence from the beginning to the end is scanned to predict any additional repeats, following the Gibbs sampler procedure^42^. Specifically, a probability position matrix is defined based on the seed, *Q*_*ij*_, which gives the probability of having in position *i*=1-MFI the particular amino acid *j*=1–20, and ranking each candidate MFI-mer relative to a background, *B*, according to: *P=*(1/MFI) *x ∑*(*Q*_*ij*_−*B*_*j*_). Note that when the seed contains only one repeat *Q*_*i*_=1, such that if *B*=0, the maximum of *P* is 1. Repeats that belong to the periodic structure will have high *P* values and will often cluster together, separated from other MFI-mers that are unrelated to the periodic structure. Thus, the cluster of MFI-mers belonging to the periodic structure is easily identified by the maximum of *ΔP* (that is, a ‘bending point', representing the large distance of the lowest ranked repeat in the cluster from other MFI-mers). If at least one member of the cluster exceeds an empirical upper threshold of 0.25, and the bending point exceeds a lower threshold of 1/MFI, then all repeats above the bending point are considered. Once repeats are added, the third step is reiterated, updating *Q* (and *P*), until no additional repeats are predicted. Note that in each such iteration, exceeding the upper threshold becomes harder. Obviously, in each step above, accumulated repeats must not overlap with each other.

The final output of the method includes the locations of the repeats in a protein. Several examples illustrating the use of this method are given in Supplementary Figs 1 and 2. As a final validating step, one may consider weakly predicted repeats, that is, those that decrease the overall IC. Testing this on the Human proteome (Supplementary Fig. 3) we found that, except for a few proteins, the IC is maximized or close to the maximum (that is, only 1–3 repeats should be removed to achieve IC maximization). In the analysis of a set of orthologues of closely related species, we assume that the number of repeats in an orthologous pair of proteins should not differ much. Hence, we use the IC maximization criterion to remove the most distant repeats from each species if this decreases the average repeat variability among the species, that is, minimizes the average of *R*_*ab*_*=|R*_*a*_–*R*_*b*_*|*/(*R*_*a*_*+R*_*b*_), where *R*_*x*_ is the number of repeats in the species *x (=a, b)*.

### Evolutionary metrics and robustness analysis

To obtain the most robust results possible when comparing relatively short sequences, we chose the least-parametrized approach, the Nei–Gojobori method^67^, to estimate selection (d*N/*d*S*) in the evolution of two sequences. Under this method, the number of non-synonymous substations per non-synonymous site (*pN*) and the number of synonymous substations per synonymous site (*pS*) are calculated, and then the d*N/*d*S* ratio is estimated with Jukes-Cantor correction^68^: d*N=−*(3/4) × log[(1−(4/3) × *pN*] and d*S=*−(3/4) × log[(1−(4/3) × *pS*]. d*N* and d*S* values are not assigned if *pN* or *pS≥0.75*, which may occur when repeats are too short or too diverged, and are discarded from analysis. Approximately 1/3 of all pairwise comparisons of repeats were discarded on the basis of this criterion. More sophisticated methods, which are parameter-rich, such as Goldman–Yang maximum-likelihood method^69^, require much longer sequences to perform adequately. Nonetheless, we use the latter to test the robustness of our main results (Supplementary Fig. 6). Robustness of d*N/*d*S* estimates is further tested with respect to the saturation of *pN* and *pS* (Supplementary Fig. 5) and the evolutionary age of proteins/repeats (Supplementary Fig. 7), and compared with d*N/*d*S* of random repeats (Figs 3 and 4). The latter are generated by shuffling the actual real coding DNA of each repeat, such that there are no stop-codons in the shuffled DNA, and translating back each repeat to a peptide. The d*N/*d*S* values of whole proteins were evaluated with the same method in each analysis (that is, Nei–Gojobori method in the main analysis and Goldman–Yang method in the robustness analysis), were compared with the values provided by Ensembl, verifying that the d*N/*d*S* distributions are distinguishable (Kolmogorov–Smirnov test), despite expected differences in the evaluation of single proteins. We consider proteins as non-repetitive if they are not compositionally ordered (that is, do not contain runs of amino acids, compositionally biased sections, nor repeats).

Binary maximum-likelihood trees describing the evolutionary relationship among repeats in a protein, or in a set of orthologous proteins (Supplementary Figs 1 and 2), were built using RaXML^70^, with the PROTGAMMA-LG substitutions model and mid-point rooting. To estimate the level of repeat homogenization (*H*) across orthologous proteins, we analyse a tree of individual repeats from orthologous proteins in a pair or a quartet of species. Clusters of homogenized repeats form subtrees where all leafs belong to the same species. The size of the largest cluster for a given species, normalized by the total number of repeats in the protein from this species, is used as the measure of homogenization.

### Data availability

All the data used in this study were downloaded from available resources such as SwissProt, Ensembl and NCBI. Proteomes and their respective coding DNA of quartets of Mammals (*Homo sapiens, Macaca mulatta, Mus musculus and Rattus norvegicus*), Fish (*Danio rerio, Astyanax mexicanus, Takifugu rubripes and Tetraodon nigroviridis*), Fly from the Drosophila genus (*D. melanogaster, D. ananassae, D. mojavensis, D. virilis*) and Plants (*Arabidopsis thaliana, Medicago truncatula, Zea mays, Oryza sativa; Japonica*) were downloaded from Ensembl (V83). Quartet sets of prokaryotes: Gammaproteobactria (*Escherichia coli, Citrobacter koseri, Erwinia amylovora, Erwinia pyrifoliae*), Firmicutes from the Bacillus genus (*Bacillus subtilis-168, B. subtilis spizizenii W23, B. licheniformis ATCC-14580 and B. licheniformis 9945A*), Actinobacteria from the Streptomyces genus (*Streptomyces bingchenggensis BCW-1, S. violaceusniger Tu-4113, S. griseus NBRC-13350, S. venezuelae ATCC-10712*) and Archaea (*Haloarcula marismortui, Haloarcula hispanica, Halorhabdus utahensis and Halorhabdus tiamatea*) were downloaded from NCBI. Accessions of human proteins analysed are provided in Supplementary Data 1. Any additional data and analysis tools are available on request from the authors.

## Additional information

**How to cite this article:** Persi, E. *et al*. Positive and strongly relaxed purifying selection drive the evolution of repeats in proteins. *Nat. Commun.* **7,** 13570 doi: 10.1038/ncomms13570 (2016).

**Publisher's note:** Springer Nature remains neutral with regard to jurisdictional claims in published maps and institutional affiliations.

## Supplementary Material

Supplementary Information Supplementary Figures 1-12 and Supplementary Methods.

Supplementary Data 1 List of 1081 human repeat containing proteins and their properties, identified by applying the computational pipeline to the Swissprot human reference proteome.

Supplementary Data 2 GO enrichment of the repeat containing proteins over a background (the entire human proteome).

Supplementary Data 3 GO enrichment of the repeat containing proteins when ranked by the horizontal *<dN/dS>*.
