# Supplementary material for: Positive and strongly relaxed purifying selection drive the evolution of repeats in proteins
Source: Nat Commun. 2016 Nov 18;7:13570. doi: 10.1038/ncomms13570 (PMC5120217; doi:10.1038/ncomms13570)
Supplement: Supplementary Information — Supplementary Figures 1-12 and Supplementary Methods. [file ncomms13570-s1.pdf]

Supplementary Information

Supplementary Figures

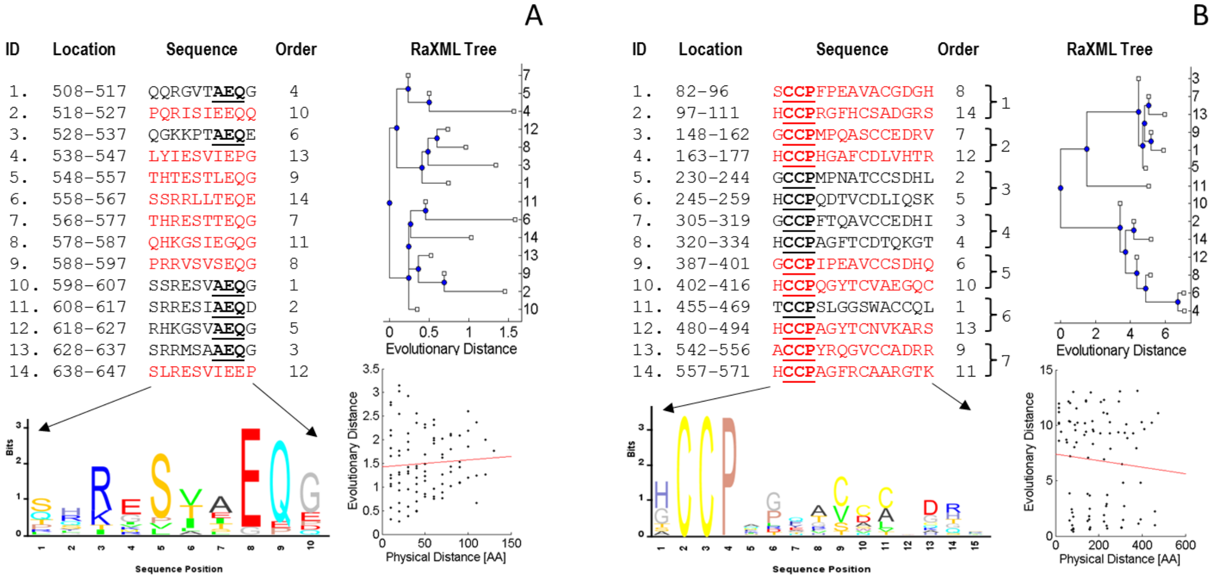

Supplementary Figure 1: Examples of repeat identification by the computational pipeline shown in **Figure 1,** applied to single proteins. **A)** 14 tandemly repetitive repeats (ID) are identified in the 1503 amino-acid long human protein EFCAB5 (EF-hand calcium-binding domain-containing protein 5). Their location, sequence and order of accumulation by the algorithm (Order) are shown. 6 repeats (black colored) are identified as *seed* in step 2, as they are all distant from each other by a harmonic of the period length (i.e., by a multiplicity of 10AA), and are *key*-aligned (i.e., by the triplet AEQ, which recurs most at interval of 10AA). Additional 8 repeats are identified by step 3 (the PPM-based predictor that scans through the entire protein). Repeats maximum likelihood tree is shown, as well as the relationship between the physical distance and evolutionary distance of all repeat pairs, showing no significant correlation in this case (Spearman = 0.094, *P*-value = 0.37). There is no annotation of repeats in SwissProt. **B)** Similarly, 14 repeats are identified in the 593 amino-acid long human protein GRN (Granulins), organized in pairs, which recur in tandem (except for pair #6). Pairs are unequally distant from each other leading to an overall partial tandem recurrence. These 7 pairs, each makes a 30AA peptide (two repeats of 15AA)**,** are embedded within the corresponding 7 Granulin domains/cleaved-chains annotated in SwissProt (each domain is 54AA-56AA long). Also, note that although the *key* triplet CPP is present in all repeats, the *seed* contains only 5 repeats that must be distant from each other by a harmonic of the period length (i.e., 15 amino-acid). Also here the correlation between the physical and evolutionary distance is not significant (Spearman = -0.088, *P*-value = 0.4).

Human

```
1  KPYKCQLCRSSFRYKGNLASHRTVHTGE
2  KPYHCSICGARFNRPANLKTHSRIHSGE
3  KPYKCETCGSRFVQVAHLRAHVLIHTGE
4  KPYPCPTCGTRFRHLQTLKSHVRIHTGE
5  KPYHCDPCGLHFRHKSQLRLHLRQKHGA
```

Mouse

```
1  KPYKCQLCRSAFRYKGNLASHRTVHTGE
2  KPYRCSICGARFNRPANLKTHSRIHSGE
3  KPYKCETCGSRFVQVAHLRAHVLIHTGE
4  KPYPCPTCGTRFRHLQTLKSHVRIHTGE
5  KPYHCDPCGLHFRHKSQLRLHLRQKHGA
```

Macaque

```
1′ KPYKCQLCRSSFRYKGNLASHRTVHTGE
2′ KPYHCSICGARFNRPANLKTHSRIHSGE
3′ KPYKCETCGSRFVQVAHLRAHVLIHTGE
4′ KPYPCPTCGTRFRHLQTLKSHVRIHTGE
5′ KPYHCDPCGLHFRHKSQLRLHLRQKHGA
```

Rat

```
1′ KPYKCQLCRSAFRYKGNLASHRTVHTGE
2′ KPYRCSICGARFNRPANLKTHSRIHSGE
3′ KPYKCETCGSRFVQVAHLRAHVLIHTGE
4′ KPYPCPTCGTRFRHLQTLKSHVRIHTGE
5′ KPYHCDPCGLHFRHKSQLRLHLRQKHGA
```

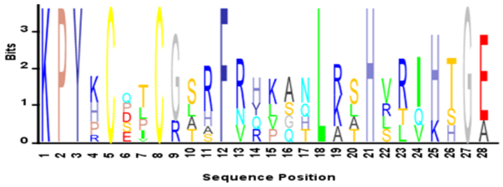

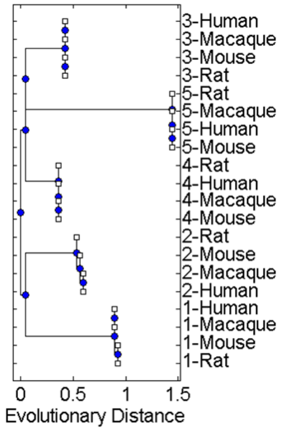

Human-Macaque

```
         K   P   Y   K   C   Q   L   C   R   S   S   F   R   Y   K   G   N   L   A   S   H   R   T   V   H   T   G   E
     1  AAA|CCC|TAT|AAG|TGT|CAG|CTG|TGC|CGG|TCT|TCG|TTC|CGC|TAC|AAG|GGC|AAC|CTT|GCC|AGT|CAT|CGT|ACA|GTG|CAC|ACA|GGG|GAA
(V)  1′ AAG|CCC|TAT|AAG|TGT|CAG|CTG|TGC|CGG|TCT|TCA|TTC|CGC|TAC|AAG|GGC|AAC|CTG|GCC|AGT|CAC|CGC|ACA|GTG|CAC|ACA|GGG|GAA (5)

         K   P   Y   H   C   S   I   C   G   A   R   F   N   R   P   A   N   L   K   T   H   S   R   I   H   S   G   E
(H)  2  AAG|CCT|TAC|CAC|TGC|TCA|ATC|TGC|GGA|GCC|CGT|TTT|AAC|CGG|CCA|GCA|AAC|CTG|AAA|ACG|CAC|AGC|CGC|ATC|CAT|TCG|GGA|GAG
(V)  2′ AAG|CCT|TAC|CAC|TGC|TCA|ATC|TGC|GGA|GCC|CGT|TTT|AAC|CGG|CCA|GCA|AAC|CTG|AAA|ACG|CAC|AGC|CGC|ATC|CAT|TCA|GGA|GAG (1)

         K   P   Y   K   C   E   T   C   G   S   R   F   V   Q   V   A   H   L   R   A   H   V   L   I   H   T   G   E
(H)  3  AAG|CCG|TAT|AAG|TGT|GAG|ACG|TGC|GGC|TCG|CGC|TTT|GTA|CAG|GTG|GCA|CAT|CTG|CGG|GCG|CAC|GTG|CTG|ATC|CAC|ACC|GGG|GAG
(V)  3′ AAG|CCC|TAT|AAG|TGT|GAG|ACG|TGC|GGC|TCA|CGC|TTT|GTA|CAG|GTG|GCA|CAT|CTG|CGG|GCG|CAC|GTG|CTG|ATC|CAC|ACG|GGG|GAG (3)

         K   P   Y   P   C   P   T   C   G   T   R   F   R   H   L   Q   T   L   K   S   H   V   R   I   H   T   G   E
(H)  4  AAG|CCC|TAC|CCT|TGC|CCT|ACC|TGC|GGA|ACC|CGC|TTC|CGC|CAC|CTG|CAG|ACC|CTC|AAG|AGC|CAC|GTT|CGC|ATC|CAC|ACC|GGA|GAG
(V)  4′ AAG|CCC|TAT|CCT|TGC|CCT|ACC|TGC|GGA|ACC|CGC|TTC|CGC|CAC|CTG|CAG|ACC|CTC|AAG|AGC|CAC|GTT|CGC|ATC|CAC|ACC|GGA|GAG (1)

         K   P   Y   H   C   D   P   C   G   L   H   F   R   H   K   S   Q   L   R   L   H   L   R   Q   K   H   G   A
(H)  5  AAG|CCT|TAC|CAC|TGC|GAC|CCC|TGT|GGC|CTG|CAT|TTC|CGG|CAC|AAG|AGT|CAA|CTG|CGG|CTG|CAT|CTG|CGC|CAG|AAA|CAC|GGA|GCT
(V)  5′ AAG|CCT|TAC|CAC|TGC|GAC|CCC|TGT|GGC|CTG|CAT|TTC|CGG|CAC|AAG|AGT|CAA|CTG|CGG|CTG|CAT|CTG|CGC|CAG|AAA|CAC|GGA|GCT (0)
```

Supplementary Figure 2: Horizontal vs vertical evolution of repeats in the zinc-finger containing gene, BCL6B - B-cell CLL/lymphoma 6, member B. 5 tandemly repeats are identified in each of the 4 mammalian species. Numbers indicate the order in which the repeat appear in each species. Each of the 5 repeats, in each species, overlaps the corresponding annotated zinc-fingers. Sequence logo of all 20 repeats, from all 4 species, and the repeats maximum-likelihood tree indicate that each repeat is highly conserved among species, in contrast to the substantial horizontal divergence of repeats within each species. **Below**, a further inspection of each of the repeats in the human-macaque orthologous pair is provided, where both proteomic and coding DNA of each repeat are shown. **Vertically**, pairs of repeats are fully conserved, i.e. 1=1', 2=2'…5=5' (repeats amino-acid sequence is shown on the top of each orthologous pair), such that there are only non-synonymous substitutions, marked in red on the lower coding DNA of each pair. Obviously *dN/dS* ratio of this vertical evolution is 0 (fully conservation). **Horizontally**, we demonstrate the case of taking the first repeat (1) as a reference, i.e., variations between 1-2, 1-3, 1-4, 1-5 are shown, marked in blue. Bold and underlined amino-acids indicate cases where in at least 2 repeats the amino-acid is the same (i.e., more conserved). The average <*dN/dS*> ratio of all pair comparisons in this horizontal evolution, averaged over the two proteins of the two species is 0.42.

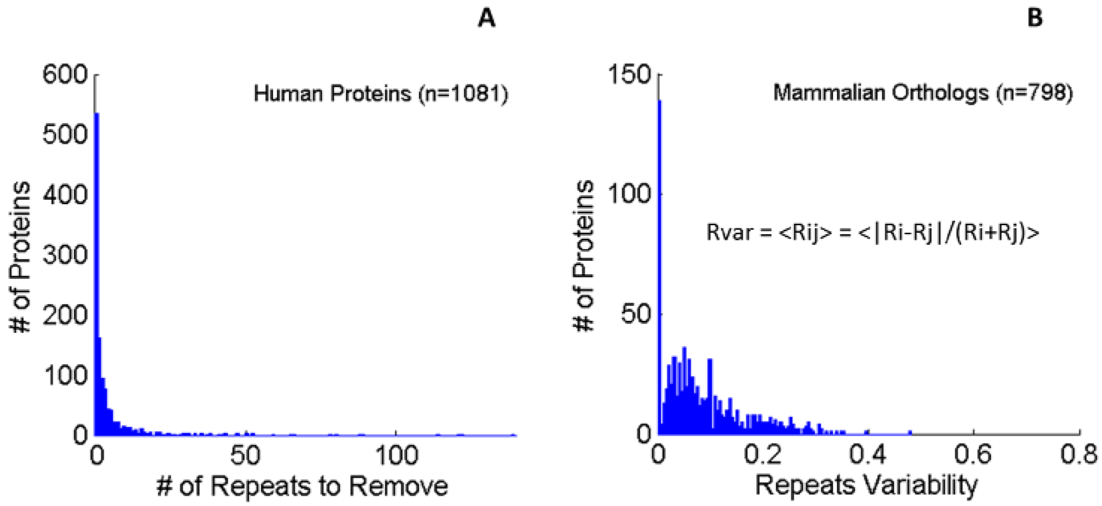

Supplementary Figure 3: IC maximization test (on human proteome) and use (on orthologous set). **A)** Histogram of the number of repeats that are needed to be removed from a protein in order to achieve maximization of the IC of the aligned repeats. More than half of the proteins already achieve IC maximization, and another third contain 1-3 more diverged repeats that leads to a deviation from IC maximization. In only few proteins a substantial number of repeats need to be removed to achieve IC maximization. In the absence of any additional information, distant repeats are considered in the analysis, because deviation from IC maximization does not mean that sequences repeats are not part of recurring phenomenon in the protein. **B)** When a set of orthologous proteins of closely related species are processed simultaneously, here the set of mammals quartet (human, macaque, mouse and rat) one can safely assume that the number of repeats in the orthologs need not vary significantly. Hence, in this case the IC maximization criterion is used to remove repeats to minimize the repeat variability (*Rvar*, see **Methods**). The resulting repeat variability is shown.

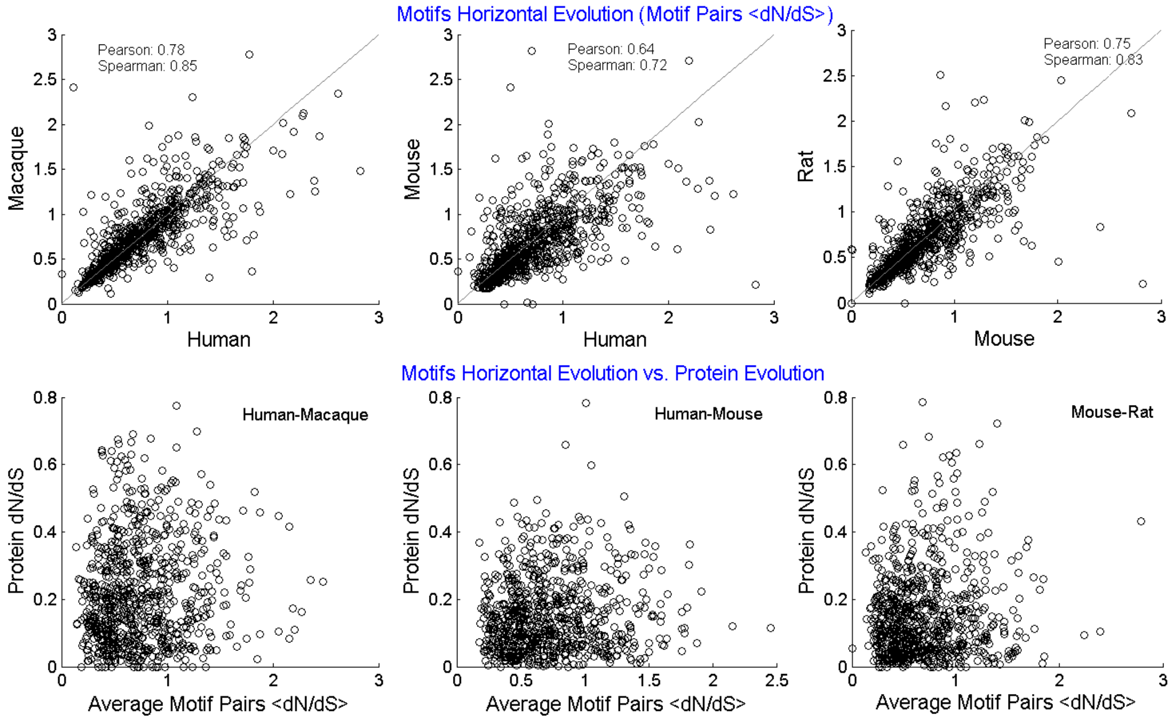

Supplementary Figure 4: Horizontal evolution of repeats *vs* protein evolution in the mammal species quartet. **Upper panel)** the relationship of the horizontal evolution of repeats (i.e., average of all pair comparisons within a protein, *<dN/dS>*) between a species pair, demonstrated for: Human-Macaque, Human-Mouse and Mouse-Rat. This high correlation allows one to take the average of the *<dN/dS>* in each species in order to assign a single value for the repeats horizontal evolution for a pair of orthologous proteins. The later can then be compared with selection acting on the full-length proteins. **Lower Panel)** the relationship between selection in the horizontal evolution of repeats (average of *<dN/dS>* across the orthologous proteins) and the *dN/dS* of the corresponding orthologous proteins, show for the same species pair in the upper panel. The lack of correlation indicates a different mechanism of selection acting horizontally the repeats than the one acting vertically on the proteins.

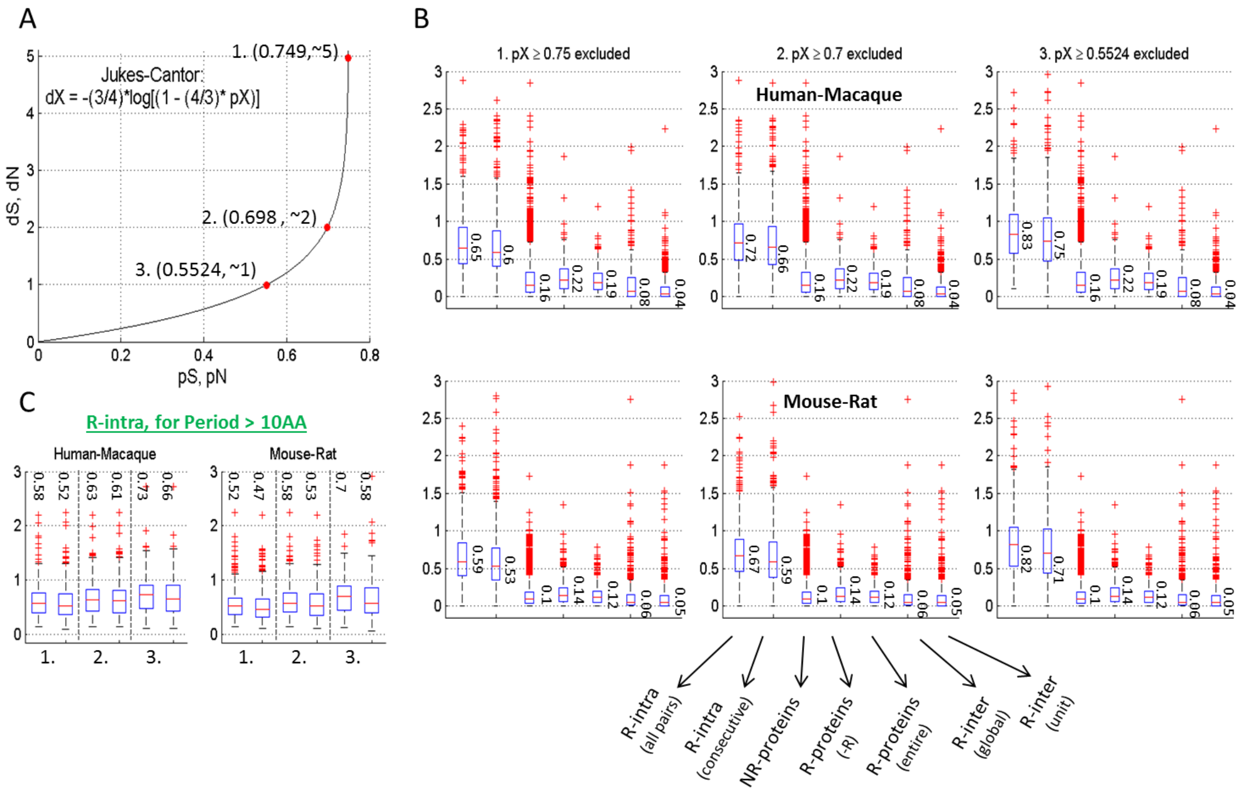

Supplementary Figure 5: Robustness to *pS*, *pN* saturation and thresholds. The analysis reported in the main text (**Figure 5**) is based on all valid repeat pair comparisons ($pX < 0.75$, where $x=S, N$), according to Jukes-Cantor formula (see **Methods**). Nonetheless, possible biases in the distribution of pX toward the saturation point (0.75), may put in question the significance of these results, because *dN/dS* may vary substantially around this point. Vertically, repeats are highly conserved (i.e., their *pX* distributions are low), hence, this possibility concerns mainly the repeat horizontal evolution. **A)** The test is whether by applying more restrictive thresholds (1-3, red dots**)** the large difference between the <*dN/dS*> of repeat horizontal (**R-intra**) vs. vertical (**R-inter**) evolution is maintained or disappears. **B)** We repeated the analysis for these increasingly restrictive thresholds (1-3). Medians of the distributions are shown in each subfigure. Comparing B-D, we observe that not only that the repeat horizontal <*dN/dS*> does not decrease for more restrictive thresholds of *pX*, it is even slightly increasing. As expected, there is no effect on the dN/dS ratio of neither protein evolution nor repeat vertical evolution. **C)** The two distributions of repeats horizontal <*dN/dS*> (all pairs and consecutive pairs) are shown for repeats whose length is larger than *10AA*, for the three tested thresholds. The increase in <*dN/dS*> vanishes. We conclude that the difference between repeats horizontal and vertical evolution is largely maintained, for reasonable choices of *pX* thresholds, and moreover, repeats longer than *10AA* are free of possible biases associated with *pX* saturation.

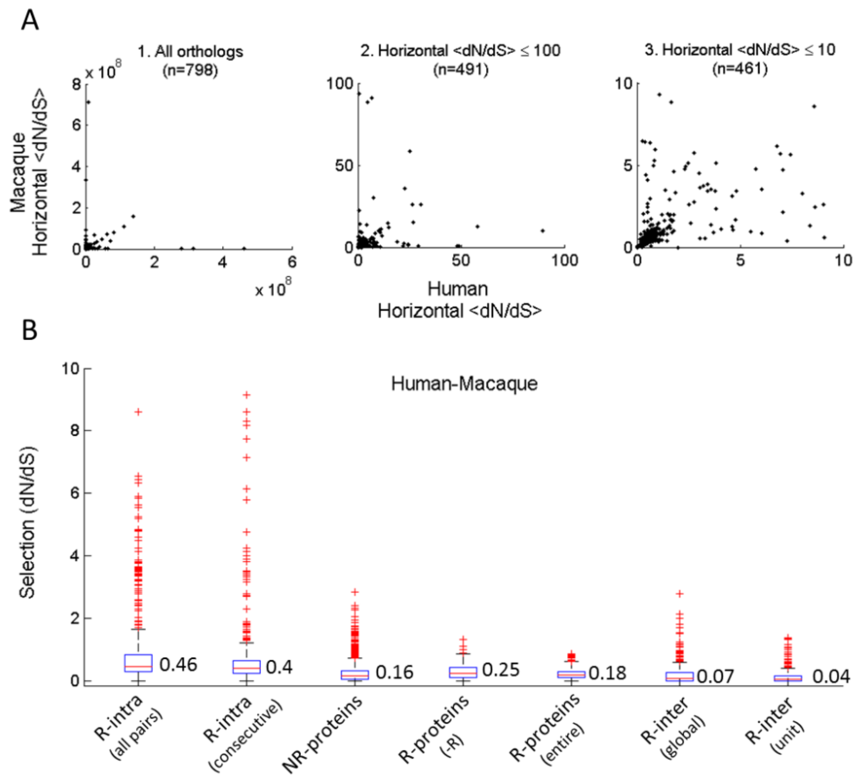

Supplementary Figure 6: Robustness test using the Goldman-Yang maximum likelihood method for estimating *dN/dS* ratios. The analysis reported in the main text (**Figure 5**) is based on the Nei-Gojobori method. As explained in Methods, this is because it is the least-parametrized method (i.e., 1-paramater model, using of Jukes-Cantor correction), and therefore more suited in this case, where we compare short sequences. Nevertheless, and although parameter-rich methods are not expected to perform adequately, we have tested that the results presented in the main text are robust with respect to the particular method of choice for estimating *dN/dS*. We have applied therefore Goldman-Yang method to the 798 Human-Macaque set of orthologous proteins (found in the analysis of the mammalian quartet). Obviously, also here in all pair comparisons for which *pN* or *pS* ≥ 0.75 are discarded from analysis. **A)** The relationship of the horizontal <*dN/dS*> between human and macaque. The 3 subfigures correspond to increasing thresholds of the maximal horizontal <*dN/dS*>. They demonstrate that for more than a third of the proteins the method is too noisy and provides unreasonably large <*dN/dS*> values (i.e. values larger than 10-100). It also shows that the correlation of horizontal selection between the two species is worse than Nei-Gojobori (compare to, **Supplementary Fig. 4**); hence further indicating the noisiness of Goldman-Yang method. This is expected because more parameters have to be estimated based on small statistics (i.e., short sequences) **B)** Focusing on proteins whose both human and macaque horizontal selection gives reasonable values (i.e., <*dN/dS*> ≤ 10) we observe the same pattern of accelerated horizontal selection and high vertical conservation; the latter is stronger than the conservation of the respective complete proteins. This result demonstrates the robustness of the main findings with respect to the exact method used for the transformation of *pS, pN* to *dS, dN*; and that the least-parametrized methods is more suited. Note that the Goldman-Yang estimates suggest a heavier tail of positive horizontal selection than the Nei-Gojobori method.

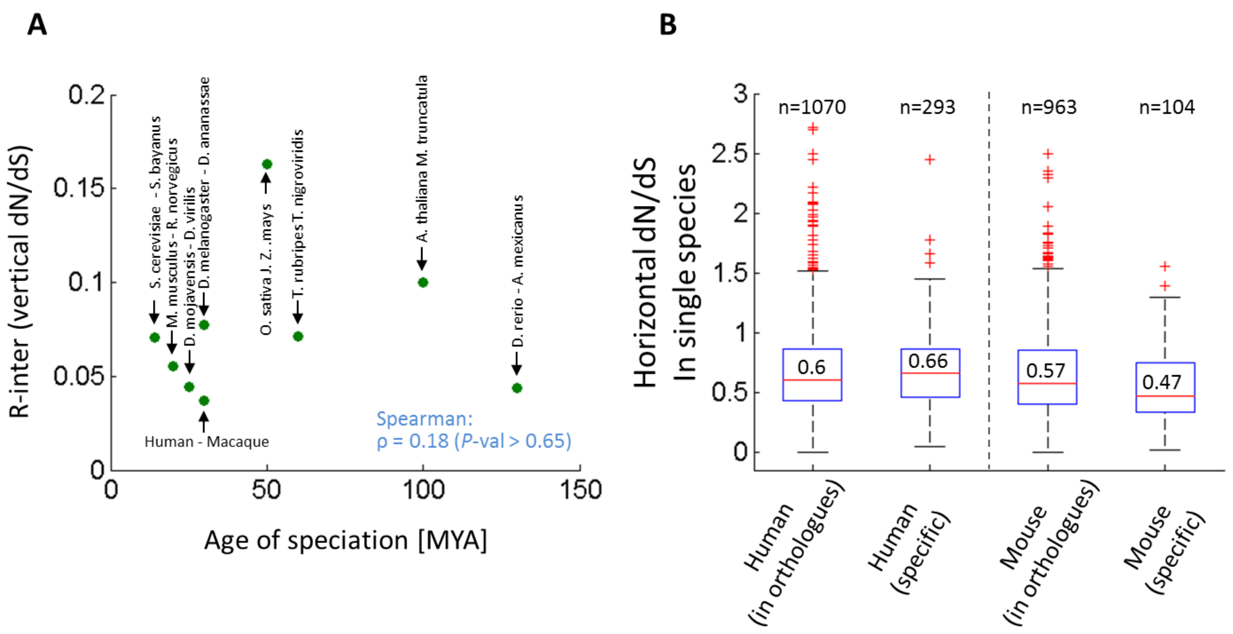

Supplementary Figure 7: Sensitivity of <*dN/dS*> distributions (and their medians) to the evolutionary age of proteins (and the repeats they encompass). **A)** Vertical evolution of orthologous repeats (R-inter medians, of **Figure 5**) as function of the evolutionary age from speciation for the 9 eukaryote species pairs (MYA = millions of years ago; data extracted from Timetree.org, see **Supplementary Fig. 9**). The lack of any detectible trend indicates that the *dN/dS* medians are not biased by the evolutionary age of the clades over an order of magnitude (14-130 MYA). **B)** Horizontal evolution of repeats within proteins, evaluated twice in human and mouse: (i) for proteins that have orthologous in any of the mammalians species (n=1070 in human, n=963 in mouse) and (ii) for species-specific proteins that do not have orthologs in any of the other mammalian species (n=293 in human, n=104 in mouse). These two groups of proteins represent different ages of the proteins and of the repeats they encompass (i.e., species-specific proteins are younger than the ones that have orthologs). Here as well, the differences between the horizontal evolution of the repeats in these two groups of proteins are insignificant, and indicate that our estimates of *dN/dS* in the horizontal evolution of repeats (relative to the vertical evolution of repeats) presented in **Figure 5** is not a consequence of the antiquity of the repeats (i.e., had originated before the common ancestor of the mammalian species quartet). Note also that in **Figure 5C**, the horizontal evolution of repeats (R-inter) in orthologous proteins is quite uniform across the diversity of eukaryote quartets (medians ~0.5-0.7), hence indirectly indicates that evolutionary age of repeats (which is unknown in this case, but obviously is different in each quartet) has no evident effect on these estimates.

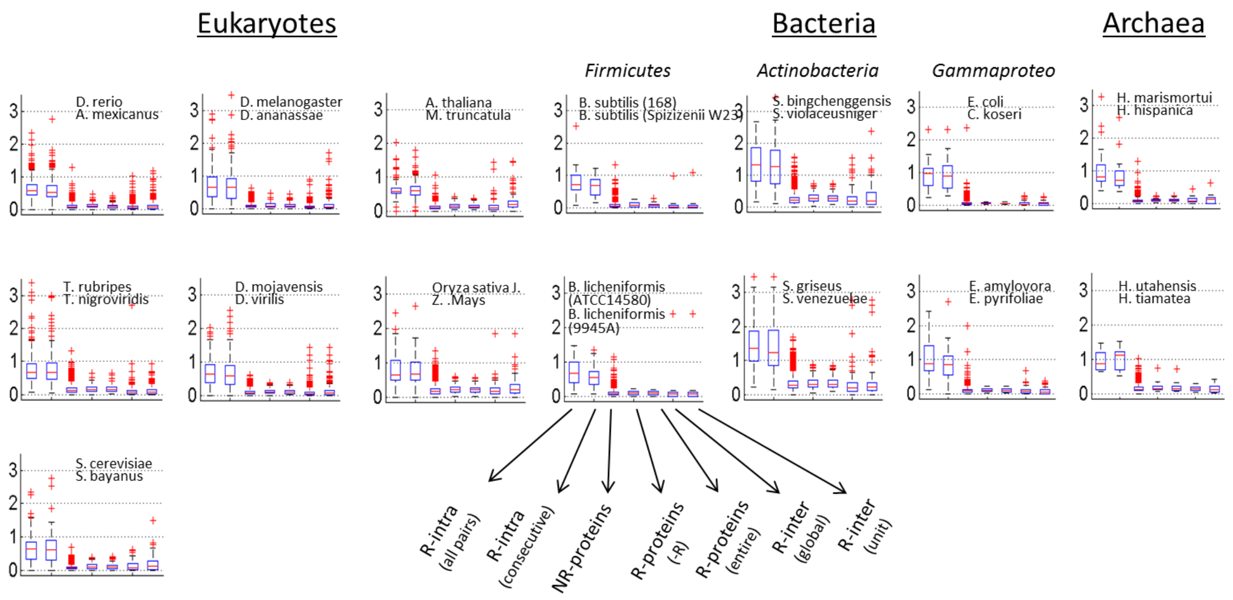

Supplementary Figure 8: The individual boxplots of the various selection measures for all the organisms studied in **Figure 5** of the main text. For mammals, see **Figure 5b** of main text.

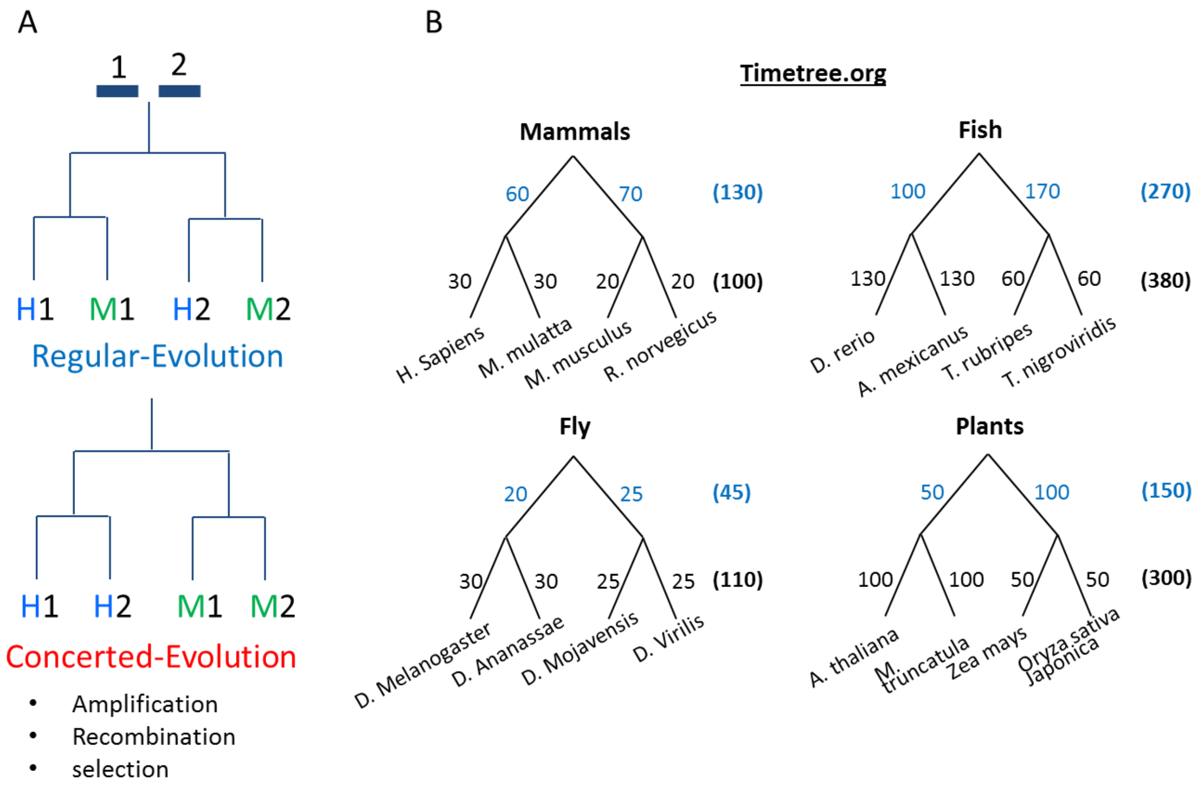

Supplementary Figure 9: Homogenization of repeats in species quartets. **A)** Similarly to gene duplication, different copies of repeats may follow regular (i.e., expected) evolution, meaning that each copy is more similar to its ortholog in a closely related species than to other copies within the same species (see example of such evolution in **Supplementary Fig. 2**). In contrast, 'concerted-evolution', i.e., copies are more similar to each other within the same species than to their orthologs, may occur by any of the three principal molecular drives: (i) amplification (i.e., an exact duplication, for example by replication slippage), (ii) recombination effects, such as unequal crossing over and (biased) gene conversion, or (iii) strong selection (either purifying or positive). **B)** We tested the homogenization level, repeat horizontal evolution and the relationship between them is all quartets examined in this study. Quartets were chosen such that the species trees are roughly symmetric. In this figure, the lengths of branches (in millions of years ago, MYA) are shown for the 4 eukaryotic quartets. Note the variability of the length of branches, but also that mammals are quite symmetric (i.e., total lengths of branches at the species and clade levels are comparable). Prokartoyic quartets were chosen such that the similarity between RNA polymerase II of two closely related species is ~95%, while between species belonging to different clades it is ~90%. These values are close to the values obtained in mammal's quartet. Homogenization is estimated by the largest cluster (node) below which all repeats belong to the species, *Hmax*. This provides a lower bound on the level of homogenization (see **Methods**). Note that concerted evolution may be identified/concluded, only if *Hmax* is high when the repeat variability (*Rvar*; **Supplementary Fig. 3**) is zero (or very low).

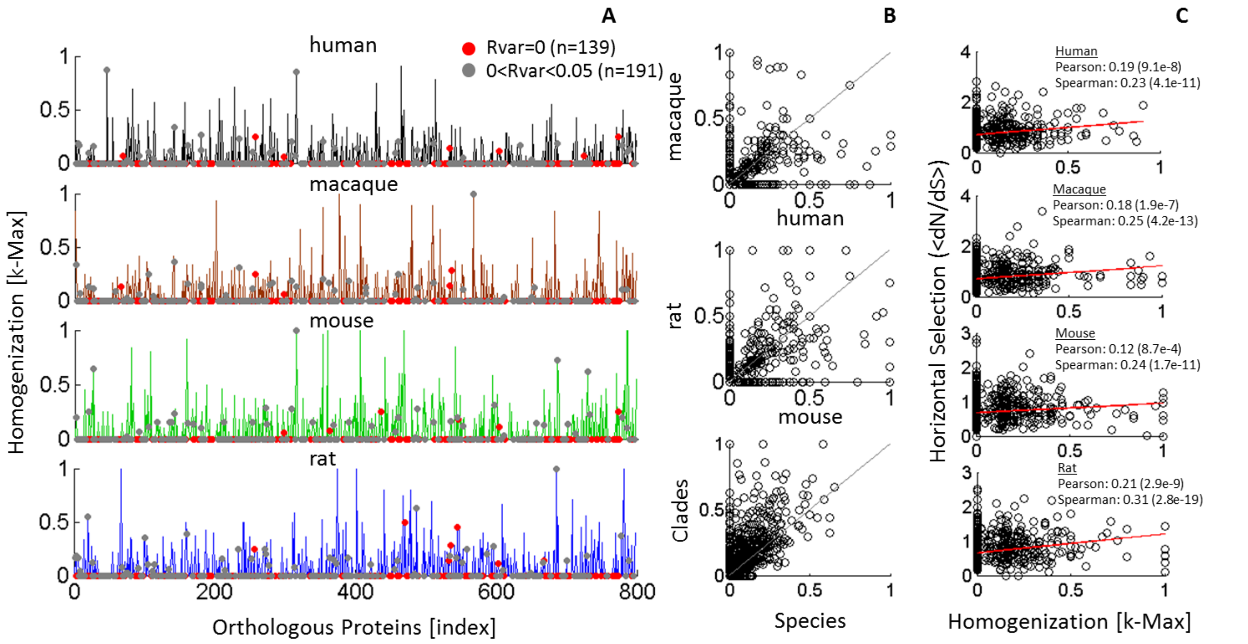

Supplementary Figure 10: Detailed analysis of the homogenization and its relationship with repeats horizontal selection in the mammalian species quartet (human, macaque, mouse and rat). **A)** The spectrum of homogenization extent, measured as the largest node below which all leafs (i.e., repeats) belong to a species, H_{max} (see **Methods**), in the four species and across the 798 repeats-containing orthologous proteins. There are 196 proteins with $H_{max}>0.25$ (in at least one of the species) and 59 proteins with $H_{max}>0.5$. Among proteins with *Rvar=0* (i.e., same number of repeats in each species), only a few proteins have significant homogenization. For larger *Rvar*, there are more proteins with significant homogenization. **This indicates that 'concerted evolution' is rare**, and that the governing regime of repeats homogenization is 'birth-death', consistent with generating mechanisms of duplication/deletion (i.e., replication slippage and recombination). When the homogenization is small repeats follow expected evolution such that each repeat is more similar to its orthologs than to its paralogs (see example in **Supplementary Fig. 2**). **B)** The correlation between the homogenization of two closely related species (top: human-macaque, middle: mouse-rat), indicating that although in many proteins repeats are homogenized in just one of the species, there's a significant fraction of proteins with highly correlated repeats homogenization. The later indicates that in these cases the mechanism of homogenization occurs at the gene level. This indicates that there are additional mechanisms involved in homogenization. Calculating H_{max} at the clade level (i.e., from the repeat ML trees where the leafs are now primates or rodents) and testing the relationship between the average H_{max} across species and the average H_{max} at the clade level we find that at the clade level repeats are more homogenized, hence, indicating (again) that repeats originated from more similar paralogous repeats in each of the common ancestors, and later diverged during evolution. Grey lines represent 1:1 linear relationship. **C)** The correlation between repeats horizontal selection and H_{max}. As shown, there is a weak but significant positive correlation (Linear regression depicted by red lines; Spearman and Pearson correlation coefficients and their corresponding *P*-values are shown). This indicates that selection also contributes to homogenize the repeats. This is expected from the strong horizontal divergence (and the weak vertical divergence) hence, positively selected mutations propagate through and eventually become fixed in the population.

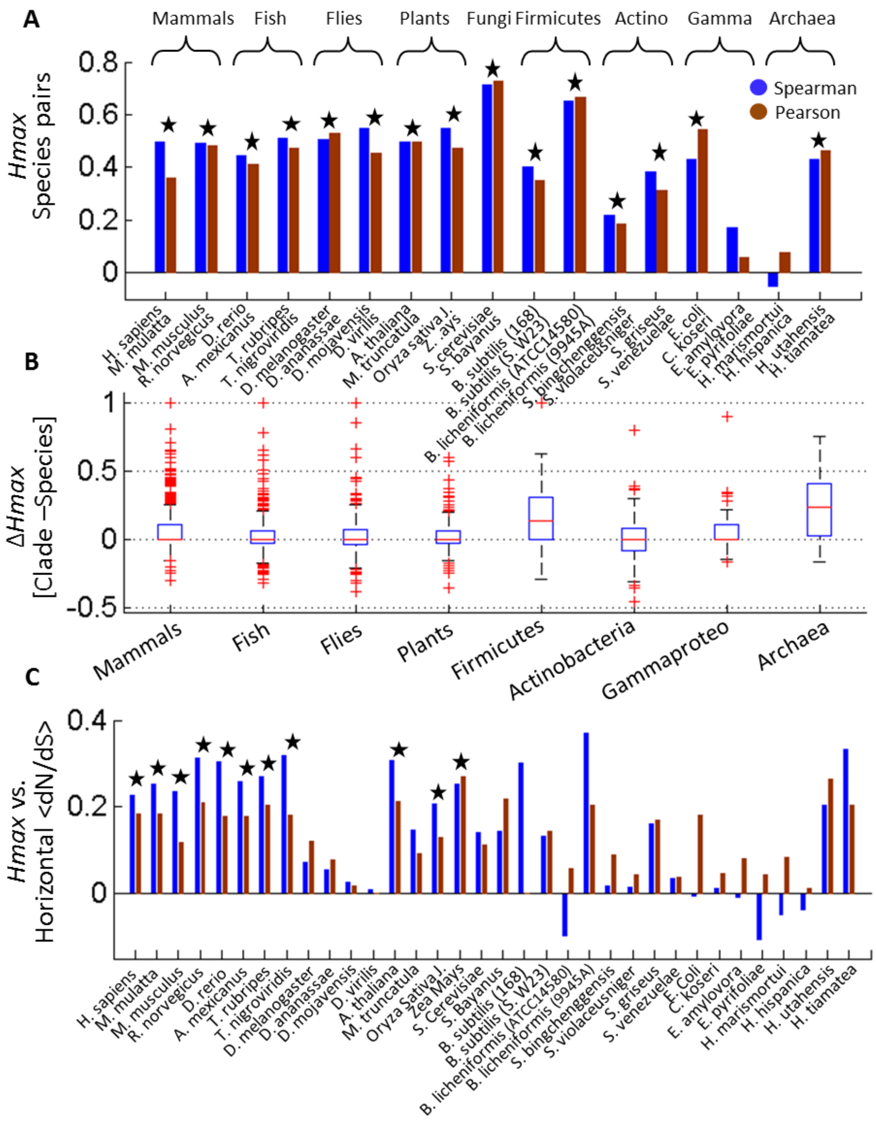

Supplementary Figure 11: Homogenization and selection across the tree-of-life, from mammals to archaea. We applied the analysis described in **Supplementary Fig. 10** to all species quartets and pairs. **A)** The correlation coefficients obtained by comparing between the homogenization levels of each two closely related species in each clade (as in **Supplementary Fig. 10B,** top and middle). Stars indicate cases where both Spearman and Pearson correlation are significant (*P*-value < 0.05). In most cases this correlation exits and is significant. **B)** Distribution of the difference between the average homogenization at the clade level and the average homogenization at the species level (see **Supplementary Fig. 10B**, bottom). Tendency towards positive values indicate that repeats are more homogenized at the clade level. Except for *Actinobacteria*, this is the case for all taxa. **C)** Correlation coefficients obtained by comparing homogenization levels and repeats horizontal selection (as in **Supplementary Fig. 10C**). Stars indicate cases where both Spearman and Pearson correlation are significant (*P*-value < 0.05). The correlation is significant only in Eukaryotes, and more specifically, in vertebrates and plants. This indicates the possible propagation of beneficial mutations (i.e., positively selected) to homogenizing the repeats.

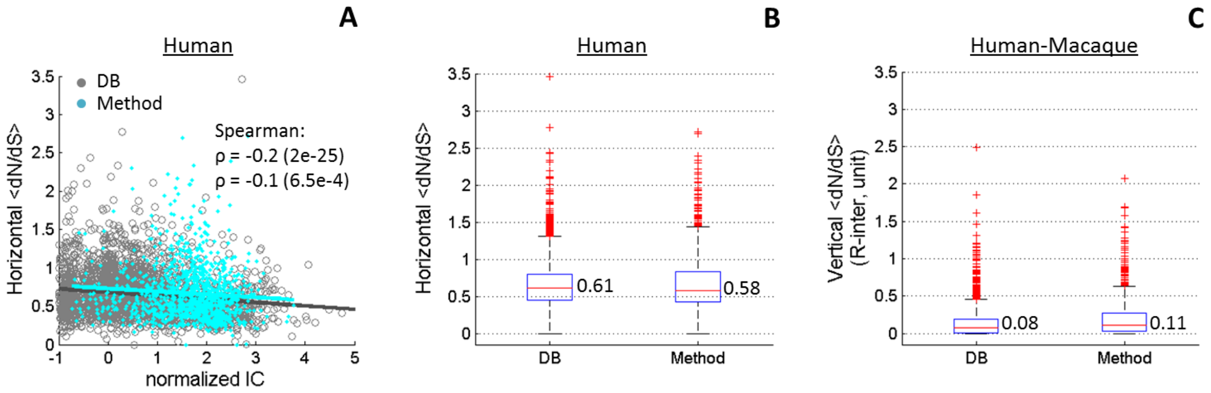

Supplementary Figure 12: Horizontal and vertical evolution of repeats as a function of the *IC* for repeats annotated in Swissprot (DB) and repeats identified with our method (Method). We extracted all the repeats annotated in Swissprot and the repeats identified with our method (see **Figure 1B)**, mapped each protein and repeat to their orthologs in macaque and estimated the *dN/dS* values for horizontal and vertical evolution in these two sets. **A)** Horizontal *<dN/dS>* as a function of *IC*. The DB-set contains many low *IC* repeats and the Method-set contains mostly high *IC* repeats. In both sets, a weak but significant negative correlation is observed between the horizontal selection and *IC*, suggesting that highly diverged repeats evolve under weaker selective constraints. Note that at low *IC* the horizontal *<dN/dS>* is still mostly < 1. However, because the strength of selection could be estimated only for pairs of repeats with *Ps* and *Pn* < 0.75 (see **Methods**), and this fraction drops from 70% in the Method-set to less than 40% in the DB-set, the selection values obtained for low *IC* repeats are likely to be underestimates. Therefore, for very low *IC* repeats, such as the ones identified by HHrepID algorithm (e.g., TIM barrels), the evolutionary regime is closer to neutrality (i.e., horizontal *<dN/dS>* ~ 1), while being conserved vertically between orthologs (see panel C). **B)** Nonetheless, the horizontal *<dN/dS>* distributions are closely similar in the DB and Method sets, and the medians of these distributions are robust estimates that are only weakly affected by the correlation demonstrated in A. This robustness of the selection estimates indicates that the horizontal *<dN/dS>* distributions analyzed in this work fairly represent a wider spectrum of repeats, including many low *IC* repeats. **C)** Vertical selection of repeats (R-inter, unit-based) in Human-Macaque orthologs is also robust, indicating strong conservation of most of the repeats, including those with low *IC*.

Supplementary Methods

The computational pipeline described under Methods and illustrated in **Figure 1** can be applied to a single protein or a set of orthologous proteins. Nonetheless, several specialized features are implemented in each step of the algorithm to ensure that the algorithm performs sufficiently well in a single protein analysis but also allows for leveraging on the larger amount of information that can be extracted from a set of orthologs.

In the first step, when a single protein is analyzed we demand that there is at least one *FT* which recurs within the *MFI* at least 3 times, to ensure that the significance of the periodic structure is high. However, when analyzing a set of orthologous proteins, the frequent-triplets (*FTs*) statistics is based on the complete distribution of all *FTs* in all proteins. This larger statistics allows for a more reliable estimation of the period length (i.e., *MFI*) and the *key* triplets which recur most within the period across all species. This facilitates the identification of a periodic structure and the repeats composing it, and resolves some scenarios that otherwise would be missed by single protein analysis, such as: (i) when the periodic structure is 'masked' (e.g., by a run of amino-acid) in some species, or (ii) when the *FT* statistics is too weak and misses the existence of a periodic structure (e.g., *MFI* is not defined in some species but significant in other species), or (iii) when *MFI* is different among species, for example, when $MFI=10$ in one species but $MFI=20$ in another. Such cases can occur due to differences in substitutions rate between the species (e.g. rodents *vs.* primates), which often results in *MFI* of one species being the harmonic of another species. Hence, when a set of orthologous proteins is analyzed one concludes a single *MFI* to be used in the analysis of all proteins, which not surprisingly is usually the lower harmonics. Note that nonetheless, if there is more than one periodic structure in a protein the method will detect only the more abundant one. Also, if in all proteins runs, doublets or triplets are dominant the method may miss a secondary periodic structure of larger repeats because it will identify $MFI \leq 3$.

In the second step, when analyzing a single protein we exploit cases where repeats recur purely (i.e. with 100% identity), and attempt to build a *seed* from such repeats first. This changes the set of *FT*-containing *MFI*-mers, to be analyzed. The logic is that if such repeats exist, the likelihood that they represent and are part of the periodic structure is high. Hence, we first build a *MFI*-mers set that includes only purely repeats. This is the case in **Figure 2**, where purely repeats exist and the *MFI*-mers are built based on them, leading to accumulation of repeats in clusters of identical repeats. Only when such purely repeats do not exist, we build a second set of *FT*-containing *MFI*-mers (see examples in **Supplementary Fig. 1-2**). This two-step MFI-mers set definition and processing is unnecessary when analyzing a set of orthologous because of the large statistics of FTs distribution. Then, relying on identically repeats is actually disadvantage because they may exist in parallel to a periodic structure which is the more dominant one, but for a single protein analysis this is the best first choice. In any scenario, proteins are always analyzed independently in this step: i.e., once the *key* and *MFI* are determined from *FTs* statistics, a *seed* is identified in each protein separately and independently.

In the third step, proteins are analyzed similarly, i.e., once the *seed* of each protein is identified, the probability position matrix (PPM) based predictor is applied to each protein, scanning it from beginning to end. Therefore, it is possible that repeats may be 'shifted' in one species relative to those identified in another species. To correct for this shift, we seek to identify the exact location of repeats in each species that maximizes the information content (IC) once all repeats in all species are aligned, and shift the

repeats in each species accordingly. This is done in two steps: (i) we identify the species whose repeats have the largest IC, and set it as a reference species, and then (ii) for each non-reference species we explore each possible shift (i.e., from *–MFI+1* to *MFI-1*, with steps of one amino-acid) and estimate the IC (of all repeats in the species) that would result following such a shift. The shift that maximizes the IC of all repeats of the two species then sets the shift to be applied to the non-reference species. Obviously, at the end of this correction all repeats in all species at best aligned relative to the reference species. An example of the final output of the analysis of a set of orthologous proteins is shown in **Supplementary Fig. 2**.
